# SMMILe enables accurate spatial quantification in digital pathology using multiple-instance learning

**DOI:** 10.1038/s43018-025-01060-8

**Published:** 2025-11-19

**Authors:** Zeyu Gao, Anyu Mao, Yuxing Dong, Hannah Clayton, Jialun Wu, Jiashuai Liu, ChunBao Wang, Kai He, Tieliang Gong, Chen Li, Mireia Crispin-Ortuzar

**Affiliations:** 1 Department of Oncology, University of Cambridge, Cambridge, UK; 2 CRUK Cambridge Centre, University of Cambridge, Cambridge, UK; 3 School of Computer Science and Technology, Xi’an Jiaotong University, Xi’an, China; 4 School of Computer Science, Northwestern Polytechnical University, Xi’an, China; 5 Department of Pathology, First Affiliated Hospital of Xi’an Jiaotong University, Xi’an, China; 6 Saw Swee Hock School of Public Health, National University of Singapore, Singapore, Singapore

**Keywords:** Cancer imaging, Tumour biomarkers, Tumour heterogeneity, Cancer, Computational biology and bioinformatics

## Abstract

Spatial quantification is a critical step in most computational pathology tasks, from guiding pathologists to areas of clinical interest to discovering tissue phenotypes behind novel biomarkers. To circumvent the need for manual annotations, modern computational pathology methods have favored multiple-instance learning approaches that can accurately predict whole-slide image labels, albeit at the expense of losing their spatial awareness. Here we prove mathematically that a model using instance-level aggregation could achieve superior spatial quantification without compromising on whole-slide image prediction performance. We then introduce a superpatch-based measurable multiple-instance learning method, SMMILe, and evaluate it across 6 cancer types, 3 highly diverse classification tasks and 8 datasets involving 3,850 whole-slide images. We benchmark SMMILe against nine existing methods using two different encoders—an ImageNet pretrained and a pathology-specific foundation model—and show that in all cases SMMILe matches or exceeds state-of-the-art whole-slide image classification performance while simultaneously achieving outstanding spatial quantification.

## Abstract (web summary)

Gao et al. present SMMILe, a multiple-instance learning-based tool that leverages whole-slide images for accurate spatial quantification without compromising on classification performance, and show it outperforms state-of-the-art methods.

## Main

Spatial predictions are critical for computational pathology. For instance, pathologists assess tissue samples visually, and explainable artificial intelligence tools are expected to produce spatial maps to guide their attention^1–3^. The discovery of spatial associations between tissue phenotype and the corresponding genotype can provide biological insights^4,5^, guide biomarker discovery^6,7^ and facilitate downstream tasks such as spatially resolved sequencing^8,9^.

A key bottleneck in the development of spatially aware computational pathology models is the need for detailed spatial annotations, which are often unfeasible due to the vast scale of gigapixel images and the need for specialized domain knowledge^10^. Multiple-instance learning (MIL) has emerged as the leading learning paradigm for whole-slide imaging (WSI) analysis^11^, owing to its ability to efficiently utilize slide-level labels and the advancement of pre-trained image feature extractors, particularly self-supervised learning-based pathology-specific foundation encoders^12–18^.

The vast majority of MIL-based computational pathology models adopt a representation-based methodology with an attention mechanism to aggregate features from different regions, enabling slide-level predictions while simultaneously identifying highly discriminative tissue regions. For example, they are used in cancer screening^19^, diagnosis^20^ and even molecular marker discovery^21,22^ and treatment response prediction^23^. These approaches have been hugely successful, achieving excellent slide-level prediction performance in different pathology scenarios. However, attention maps generated by representation-based MIL approaches can only be qualitatively interpreted through manual analysis and are increasingly viewed as suboptimal for spatially aware predictions^24–26^. This limitation stems from the reliance on slide-level labels, as attention scores are not directly correlated with the slide-level labels and can be misled by confounding factors^27^. This often leads to overfitting on irrelevant regions or only a subset of discriminative areas, making it challenging for the model to infer all clinically important regions accurately^28,29^. These factors substantially hamper the utility of representation-based MIL approaches in scenarios that require accurate phenotypic descriptions beyond a simple global label^30^.

**Fig. 1:**
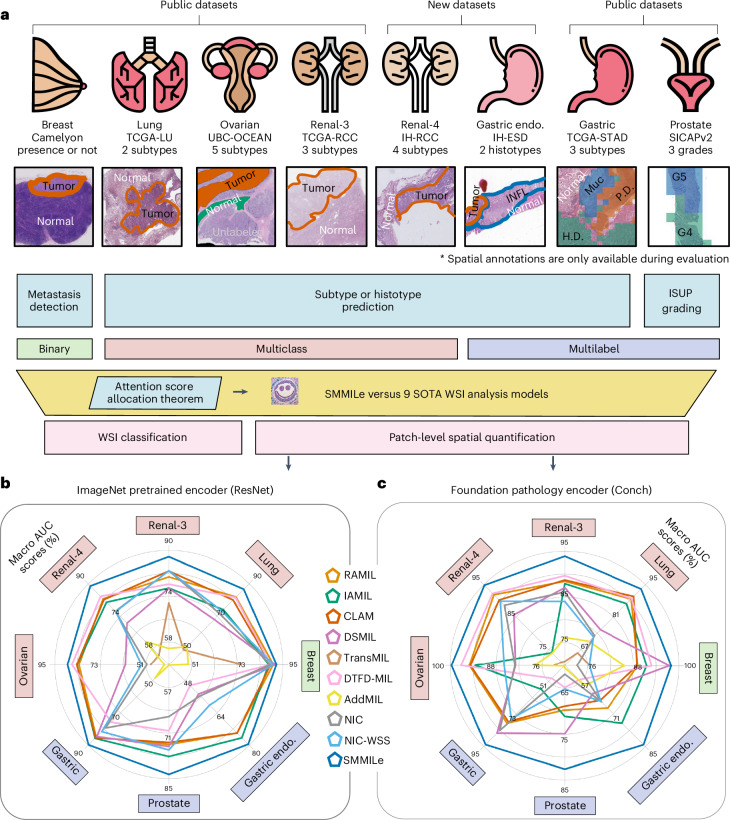
Study overview and evaluation. **a**, Study overview. The distinct attention score allocation mechanisms across different MIL methodologies are theoretically analyzed. Building on this foundation, we present SMMILe and compare it with nine SOTA WSI classification techniques across six public and two in-house datasets. In addition to WSI-level labels used for model training, these datasets contain complete or partial spatial annotations, which are used only for evaluation. For binary and multiclass classification, red represents the annotated tumor region; for multilabel classification, different colors represent different histotypes. In the Gastric Endoscopy (Endo.) dataset, INFL indicates inflammation; P.D., H.D. and Muc represent poor differentiation, high differentiation and mucinous, respectively; and in the Prostate dataset, G3, G4 and G5 represent different Gleason grades. These datasets encompass tasks such as metastasis detection, subtype prediction and International Society of Urological Pathology (ISUP) grading across six distinct cancer types. See the Methods for details on the attention score allocation theorem, SMMILe methodology and evaluation datasets. **b**,**c**, Radar plots comparing the patch-level spatial quantification performance of SMMILe and baseline methods on all evaluation datasets: Breast (*n* = 3,956,231 patches), Lung (*n* = 316,146 patches), Ovarian (*n* = 47,592 patches), Renal-3 (*n* = 290,061 patches), Renal-4 (*n* = 421,931 patches), Gastric Endoscopy (n = 117,735 patches), Gastric (*n* = 92,570 patches) and Prostate (*n* = 18,426 patches), using the ImageNet pretrained encoder (ResNet-50) and the pathology foundation model encoder (Conch), respectively. Source data

In this Report, we introduce SMMILe (Superpatch-based Measurable Multiple Instance Learning), a WSI analysis method designed to perform spatial quantification alongside WSI classification. SMMILe builds upon instance-based MIL, which stands apart from representation-based MIL by offering superior localization abilities^31^. We systematically benchmark nine well-established WSI classification methods, comparing SMMILe across three key pathology tasks: metastasis detection, subtype prediction and grading; framed as binary, multiclass and multilabel classification problems. This study spans eight datasets covering six cancer types (lung, renal, ovarian, breast, gastric and prostate; Fig. 1a), providing a comprehensive comparison (Fig. 1b,c) across diverse clinical scenarios. While classification performance is assessed, our primary focus is on evaluating the spatial quantification capabilities of each method by integrating encoders with different capabilities, including a widely used ImageNet pretrained model and a pathology-specific foundation model.

## Results

### Instance-based learning produces highly skewed attention maps

**Fig. 2:**
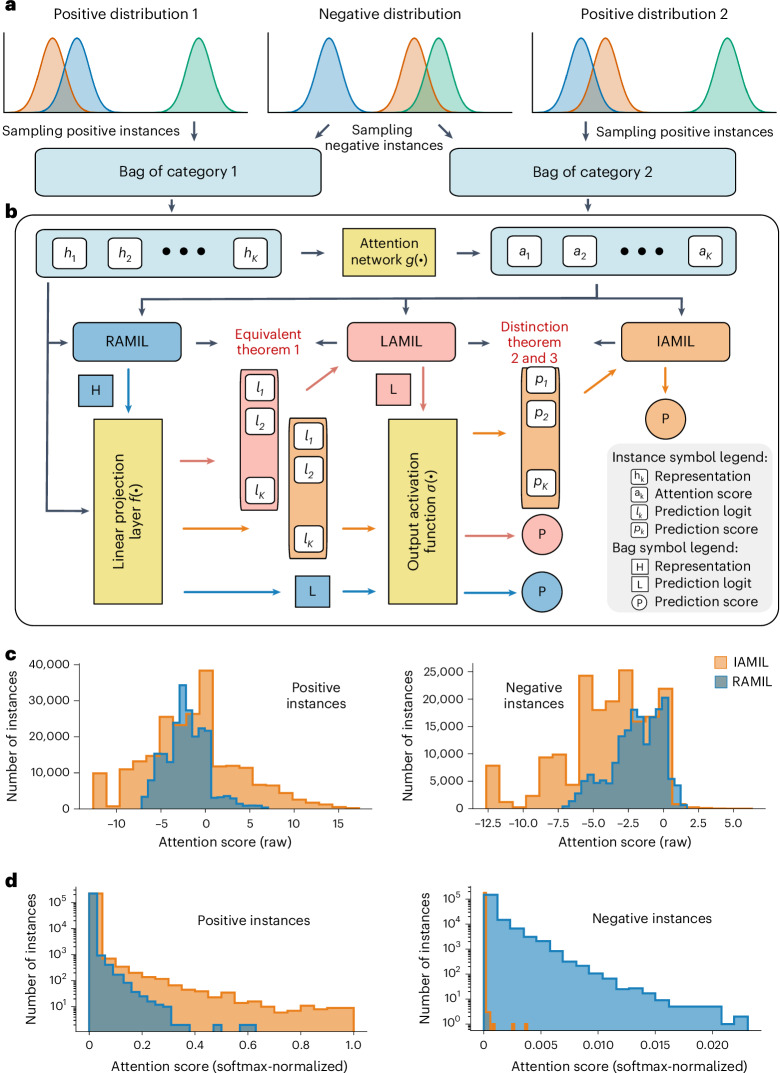
IAMIL versus RAMIL. **a**, Synthetic data generation. This synthetic MIL dataset consists of two categories. For these, we designed three types of instance distribution using Gaussian distributions: two are discriminative (positive) and one is nondiscriminative (negative). **b**, Frameworks of three attention MIL. First, all three methods calculate an attention score for each instance by the attention network. Then, based on these scores, RAMIL combines data at the representation level, LAMIL at the logit level and IAMIL at the score level. **c**, Histograms of raw attention scores for positive and negative instances. **d**, Histograms of softmax-normalized attention score for positive and negative instances (RAMIL in blue, IAMIL in orange; *n* = 100,000 instances each). Source data

Despite sharing a foundational MIL framework, instance-based (IAMIL) and representation-based (RAMIL) multiple-instance learning methods diverge in their methodologies for assigning attention scores to individual instances. Understanding these differences is critical to design a framework that has the best of both worlds. With that objective, we analyze the mathematical formulation of the different frameworks by means of three theorems (see ‘RAMIL versus IAMIL’ section in the Methods) and a targeted synthetic experiment (Fig. 2a,b and Supplementary Table 1).

**Extended Data Fig. 1:**
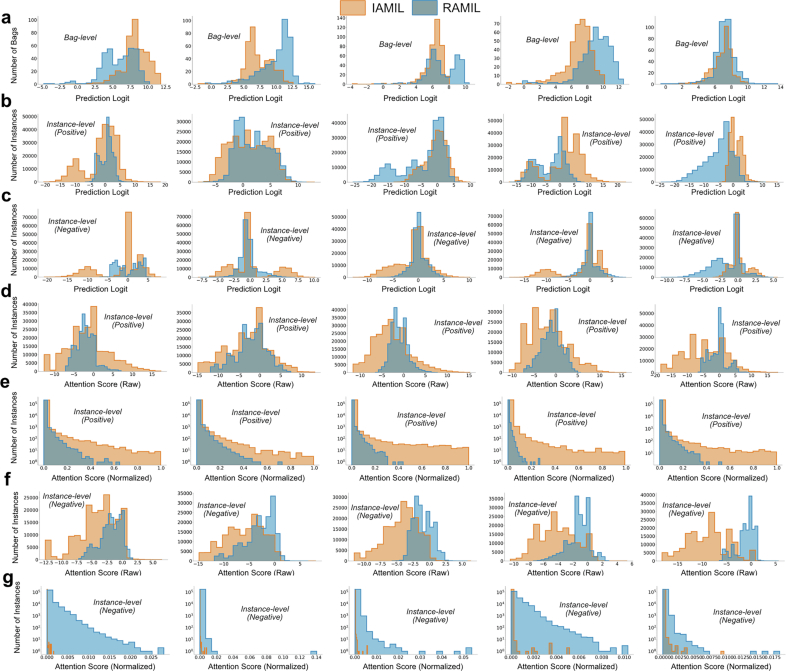
Comparison of attention score distributions for RAMIL and IAMIL across five synthetic experiments. Histograms with 20 bins from five synthetic experiments (from left to right) for RAMIL and IAMIL. **a**, The prediction logits of bags (${L}_{c}$). Since IAMIL directly outputs bag scores, we computed its bag logits inversely; **b**, The prediction logits (${l}_{i}^{c}$), **d**, the raw attention scores (${z}_{i}^{c}$), and **e**, the softmax-normalized attention scores (${a}_{i}^{c}$) for positive instances; **c**, The prediction logits (${l}_{i}^{c}$), **f**, the raw attention scores (${z}_{i}^{c}$), and **g**, the softmax-normalized attention scores (${a}_{i}^{c}$) for negative instances. The y-axes of histograms for ${a}_{i}^{c}$ in (**e**) and (**g**) are on a logarithmic scale. Source data

Our theorems indicate that IAMIL assigns lower attention scores to low-discriminative and nondiscriminative instances while allocating higher scores to highly discriminative instances, compared with RAMIL. This pattern was confirmed by our synthetic experimental results (Fig. 2c,d and Extended Data Fig. 1a–g) where IAMIL consistently assigned negative instances much lower attention scores (mostly below 0.002), whereas RAMIL often assigned scores above 0.01. Furthermore, IAMIL gave notably higher attention scores to highly discriminative instances, often twice as high as those in RAMIL (see ‘Synthetic experiment’ in the Supplementary Note for details).

Together with our theoretical findings, these synthetic experimental results also highlight the key limitation of IAMIL: it produces highly skewed attention maps, focusing only on a limited subset of high-discriminative regions within WSI, leading to a decreased recall rate for the relevant tissue regions. This observation aligns with the initial findings in ref. ^32^. Solving this critical shortcoming is the main barrier for IAMIL to achieve its potential as a superior foundational approach for accurate spatial quantification in WSIs.

### WSI classification

Building on from these results, we developed SMMILe, a superpatch-based measurable MIL method. SMMILe is designed to benefit from the spatial awareness of IAMIL and is equipped with custom modules that address the shortcomings identified previously.

**Fig. 3:**
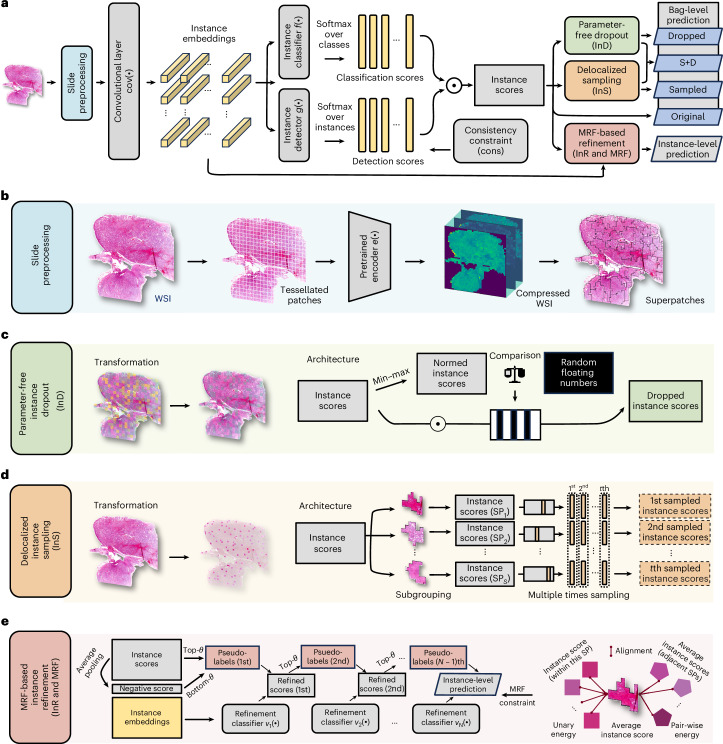
Model schema of SMMILe. **a**, Overview of SMMILe. **b**, Slide preprocessing. Each WSI is tessellated into patches and mapped into embeddings by the pretrained encoder. Then, we obtain the compressed WSI using NIC, which prepares it for the application of convolution operations. Concurrently, SMMILe forms a series of superpatches (SP; subscript *S* denotes the total number of superpatches) by oversegmentation at compressed WSI. **c**, Parameter-free instance dropout (InD), involving dropping high-discriminative instances based on their instance scores. **d**, Delocalized instance sampling (InS), involving generating multiple sub-bags by randomly selecting one instance from each superpatch in multiple rounds. **e**, MRF-based instance refinement (InR and MRF), involving training an auxiliary multistage refinement network by self-training strategy with pseudo-labels, enhancing the spatial smoothness by MRF energy constraints.

SMMILe (Fig. 3a), comprises a convolutional layer, an instance detector, an instance classifier and five modules introduced in this work: slide preprocessing (Fig. 3b), consistency constraint (Fig. 3a), parameter-free instance dropout (Fig. 3c), delocalized instance sampling (Fig. 3d) and Markov random field (MRF)-based instance refinement (Fig. 3e). The convolutional layer for the instance embeddings enhances their local receptive field. The instance detector, designed with multiple streams, identifies the significance of each instance’s embedding for different categories, facilitating multilabel classification tasks. The classifier assigns each instance’s embedding to its respective category. Ultimately, the bag-level (WSI) classification is determined by aggregating the product of detection and classification scores from all instances (patches).

We evaluated the WSI-level classification performance of SMMILe on eight distinct cancer datasets across three different categories of pathology tasks of increasing complexity (Table 1).

**Table 1:** Statistics of the datasets used in experiments

Task	Dataset	Magnification	Patch size	Category	WSIs (patients) total	WSIs (samples) annotated	Patches annotated
Binary classification	Breast (Camelyon16)	40×	512	Tumor, Normal	399	399	3,956,231
Multiclass classification	Lung (TCGA-LU)	40× (20×)	2,048 (1,024)	LUAD, LUSC	950 (851)	523	316,146
	Ovarian (UBC-OCEAN)	20×	512	CC, EC, HGSC, LGSC, MC	513	152	47,592
	Renal-3 (TCGA-RCC)	40× (20×)	2,048 (1,024)	CCRCC, PRCC, CHRCC	660 (644)	338	290,061
	Renal-4 (IH-RCC)	20×	1,024	CCRCC, PRCC, CHRCC, ROCY	563 (168)	138	421,931
Multilabel classification	Gastric Endoscopy (IH-ESD)	20×	512	Tumor, Inflammation, Normal	99	99 (286)	117,735
	Gastric (TCGA-STAD)	40× (20×)	2,048 (1,024)	High, Poor, Mucinous	339 (321)	128	92,570
	Prostate (SICAPv2)	10×	512	G3, G4, G5, Normal	153	153	18,426

‘WSIs annotated’ denotes WSIs with patch-level labels. CC, clear cell carcinoma; EC, endometrioid carcinoma; HGSC, high-grade serous carcinoma; LGSC, low-grade serous carcinoma; MC, mucinous carcinoma.

The simplest category was binary classification. Both instances and bags are categorized into only positive and negative classes (for example, presence of cancer or not). As an example, we trained the framework to detect breast lymph node metastases using the well-studied Breast (Camelyon16) dataset^33^. We also studied multiclass classification, in which instances and bags can belong to one of a number of positive categories, or to the negative class (for example, different cancer subtypes versus no cancer). As an example, we studied four different examples of cancer subtyping tasks, two of which are from The Cancer Genome Atlas (TCGA) project: Lung (TCGA-LU, non-small cell lung cancer subtyping)^34,35^, Renal-3 (TCGA-RCC, renal cell carcinoma subtyping)^36–38^, Renal-4 (IH-RCC, renal tumor subtyping) and Ovarian (UBC-OCEAN, ovarian cancer subtyping)^39,40^. Finally, we considered multilabel classification, which remains relatively unexplored in previous computational pathology literature. Here, a bag can contain instances from multiple positive categories, in addition to negative instances (for example, mixed-type tumor). We evaluated three datasets for heterogeneity tumor analysis: Gastric (TCGA-STAD, gastric cancer subtyping)^41^, Gastric Endoscopy (IH-ESD, histotype classification for endoscopic submucosal dissection specimens) and Prostate (SICAPv2, Gleason grading)^42^. All datasets were randomly divided at the patient or WSI level into five subsets to facilitate fivefold cross-validation, with the results reporting the mean and variance for each metric.

We benchmarked SMMILe against the two fundamental attention-based MIL methods (RAMIL and IAMIL), in addition to five current state-of-the-art MIL-based WSI classification methods: CLAM^43^, DSMIL^24^, TransMIL^44^, DTFD-MIL^25^ and AddMIL^28^. Moreover, we considered two neural image compression (NIC)-based WSI classification methods: the standard NIC approach^45^ and NIC-WSS^46^, an enhanced variant optimized for regional segmentation. Except for the already tessellated Prostate dataset, we processed these WSIs into patches without overlapping. Patch embeddings were extracted using two encoders for comparison. First, patch embeddings were extracted from an identical layer (the third residual block) of the ResNet-50, which had been pretrained on the ImageNet dataset. Second, we utlized patch embeddings from the foundation model Conch^47^, which was pretrained using 1.17 million pathology image–caption pairs. The two encoders were uniformly applied in turn across all baselines to ensure equitable comparison.

**Fig. 4:**
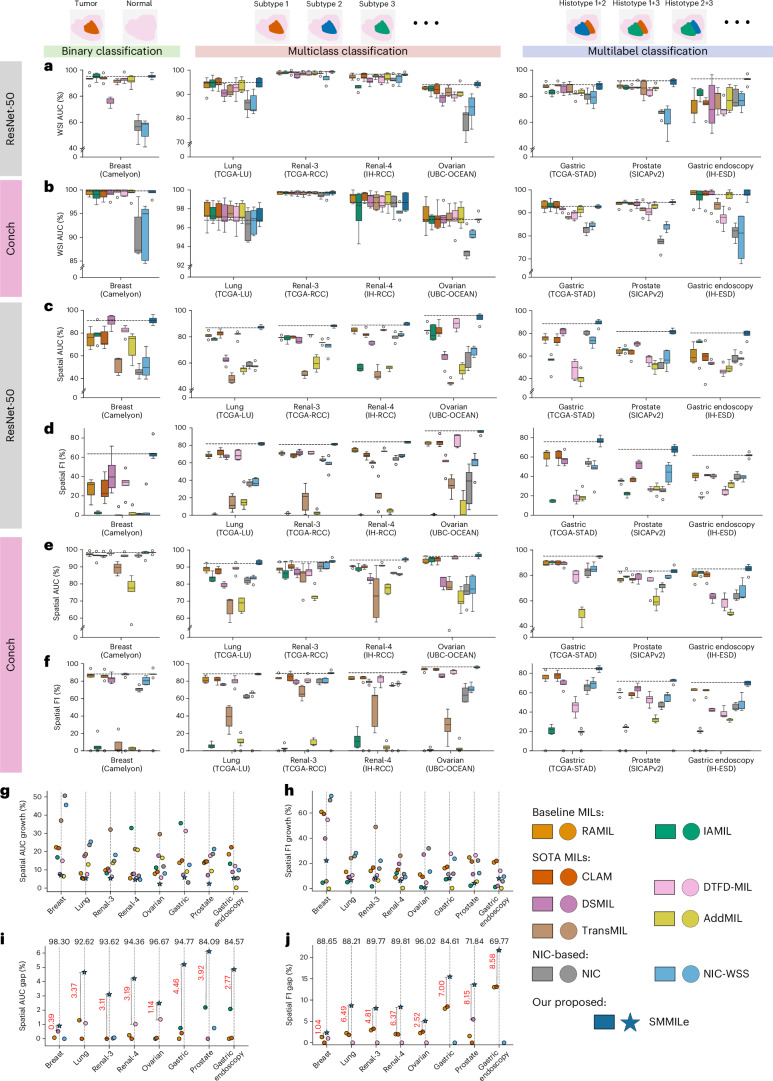
Performance of WSI classification and spatial quantification. **a**,**b**, WSI classification performance (AUC score) of all methods using the ImageNet-pretrained ResNet-50 (**a**) and the pathology foundation model Conch (**b**) as encoders. **c**,**d**, Spatial quantification performance (AUC (**c**) and F1 scores (**d**)) of all methods using the ImageNet pretrained ResNet-50 as the encoder. **e**,**f**, Spatial quantification performance (AUC (**e**) and F1 (**f**) scores) of all methods with the pathology foundation model Conch as the encoder. All box plots are based on fivefold cross-validation using independent tissue samples: Breast (*n* = 399 WSIs; 3,956,231 patches), Lung (*n* = 950 WSIs; 316,146 patches), Ovarian (*n* = 513 WSIs; 47,592 patches), Renal-3 (*n* = 660 WSIs; 290,061 patches), Renal-4 (*n* = 563 WSIs; 421,931 patches), Gastric Endoscopy (*n* = 99 WSIs; 117,735 patches), Gastric (*n* = 339 WSIs; 92,570 patches) and Prostate (*n* = 153 WSIs; 18,426 patches). Box plots show the median (center line), upper and lower quartiles (box bounds), whiskers extending to 1.5× the interquartile range, and individual points representing outliers. **g**,**h**, The improvement in spatial quantification (AUC (**g**) and F1 (**h**) scores) when switching from the ImageNet pretrained encoder to the Conch encoder. **i**,**j**, The performance gap in spatial quantification (AUC (**i**) and F1 (**j**) scores) between SMMILe and the top-4 methods with the Conch encoder, using the performance of the fourth-ranked method as the baseline, setting its value at 0 on the *y* axis for comparison. The absolute values of SMMILe’s performance are marked at the top of the figure. Also, the difference between SMMILe and the second-best method is highlighted with distance lines for clarity. All data points in **g**–**j** represent the mean values across fivefold cross-validation. Dashed lines in **a**–**f** indicate SMMILe’s performance. Source data

Using patch embeddings generated by ImageNet pretrained encoder (ResNet-50), SMMILe consistently outperforms comparison methods in WSI classification across all datasets in terms of macro Area Under the Receiver Operating Characteristic Curve (AUC) score (Fig. 4a and Supplementary Table 2). While some methods, such as IAMIL, CLAM, TransMIL and DTFD-MIL, show competitive performance on certain datasets, SMMILe achieves AUC scores of 94.11%, 90.92% and 92.75% on the Ovarian, Prostate and Gastric Endoscopy datasets, respectively, exceeding the second-best methods by margins of 2.20%, 2.90% and 11.18%. Also, the interquartile ranges of box plots (Fig. 4a) further underscore the robustness of SMMILe in WSI classification across different datasets. The two methods based on NIC (NIC and NIC-WSS) and DSMIL demonstrate suboptimal WSI classification performance on most datasets. This shortcoming originates mainly from their intrinsic design, which is intended for handling WSI embeddings generated by pretrained encoders finely tuned for discriminative features pertinent to specific domains. These results demonstrate that SMMILe can deliver superior and consistent WSI classification performance across various cancer types and pathology tasks with suboptimal patch embeddings.

Using Conch, a pathology foundation model, markedly improved the performance of all methods in WSI classification tasks (Fig. 4b and Supplementary Table 2). In binary and multiclass classification tasks, most methods achieved a macro AUC score of over 97% across all datasets, with performance approaching 100% in Breast, Renal-3 and Renal-4 datasets. Even for the more challenging multilabel classification tasks, most methods reached performance levels of 90–95%. This highlights the remarkable capability of the foundation model encoder in boosting performance across diverse downstream tasks. Although SMMILe did not perform best on every dataset, it consistently delivered competitive results comparable to the top-performing methods. Notably, methods (NIC, NIC-WSS and DSMIL) that rely on domain-specific pretrained encoders show notable improvements in WSI classification.

### Spatial quantification

Beyond the accurate classification of entire WSIs, we wanted to assess the capabilities of SMMILe for spatial quantification, that is, patch-level classification, compared with existing methods. Spatial ground truth (GT) annotations were available for eight datasets, either in their entirety (Breast, Gastric Endoscopy and Prostate datasets) or in part (Lung, RCC-3, RCC-4 and Gastric datasets). The Ovarian dataset represents a unique case, with annotations available for only a subset of patches within certain WSIs. Consequently, the evaluation of this dataset is confined to the spatial quantification of these specifically annotated patches. None of the spatial annotations was used for model training in any case.

The derivation of patch-level predictions in representation-based attention MIL baselines (RAMIL, CLAM, DSMIL, TransMIL and DTFD-MIL) relies on the raw attention scores. In the case of NIC and NIC-WSS, patch-level predictions are acquired from grad-CAM^48^ outputs, whereas, for the instance-based MIL methods (IAMIL and AddMIL), predictions are based on instance scores. SMMILe possesses an instance refinement network capable of directly generating patch-level predictions. We use accuracy, AUC, precision, recall and F1 score as the spatial quantification metrics. For multilabel tasks, where a sample contains multiple patch labels, the metrics are macro averaged.

**Extended Data Fig. 2:**
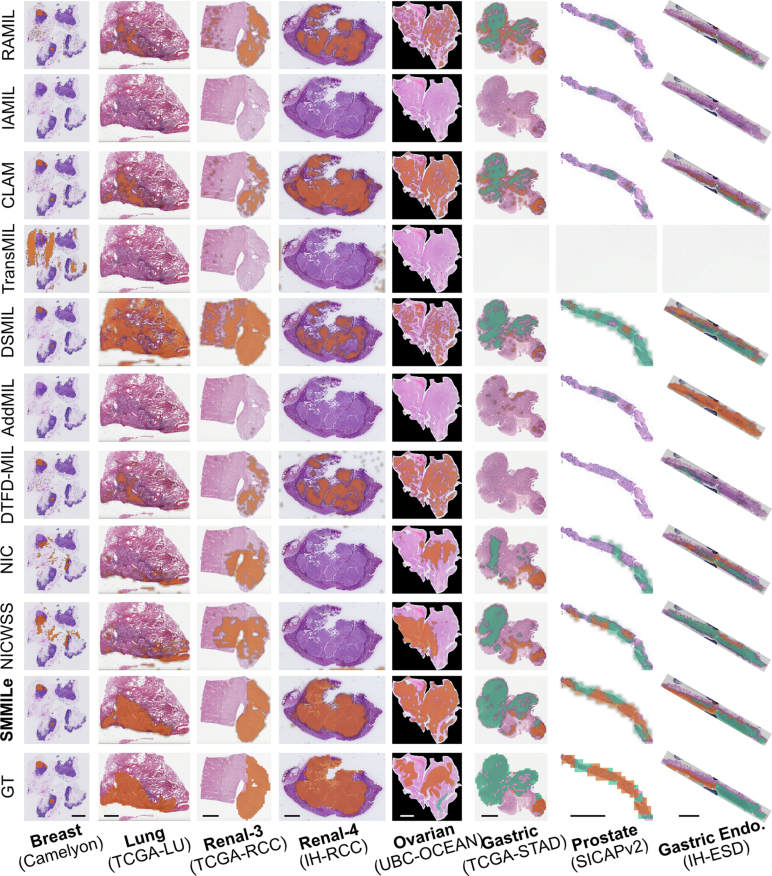
Visualization of spatial quantification results across eight datasets using the ImageNet pretrained ResNet-50 as the encoder. For all datasets, to ensure uniform display, the ground truths (GTs) are shown as patch-level masks, while the spatial quantification results are displayed as patch-level masks with Gaussian smoothing applied. For the Breast, Lung, Renal-3, Renal-4, and Ovarian cases, orange represents tumor regions, while green in the Ovarian GT indicates normal tissue. In Gastric, Prostate, and Gastric Endo. cases, different colours indicate different histotypes. In Gastric, orange, green, and blue indicate poor differentiation, high differentiation, and mucinous subtypes. In Gastric Endo., orange and green denote tumor and inflammation regions. In Prostate, G3 to G5 tumor regions are shown in orange, green, and blue. Uncoloured regions are classified or annotated as normal. Scale bar, 4 mm (applies to all columns).

Across all evaluated datasets, SMMILe surpasses other methods by substantial margins with the ImageNet pretrained encoder, ResNet-50 (Fig. 4c,d and Supplementary Tables 3–7). It achieves spatial AUC scores that either exceed or approach 90% in nearly all datasets. Exceptions are the Prostate and Gastric Endoscopy datasets, which are characterized by extremely imbalanced patient distribution and a limited number of patients. Despite this, SMMILe still demonstrates strong performance with spatial AUC scores around 80%. For the Breast dataset, due to the highly imbalanced patch distribution (with only a few tumor regions per metastasis case), several methods (IAMIL and DSMIL) achieve high accuracy and macro AUC scores. This is because these instance-based methods have a natural advantage in detecting a small number of positive instances. However, while SMMILe surpasses the second-best method (DSMIL) by only about 1% in spatial AUC, it substantially outperforms DSMIL by 23.56% in spatial F1 score. Specifically, SMMILe outperforms the second-best methods by over 20% on the Breast and Gastric Endoscopy datasets, by more than 15% on the Gastric and Prostate datasets and by nearly 10% on the Lung, RCC-3, RCC-4 and Ovarian datasets, in terms of spatial F1 score. The visualization of spatial quantification using the ResNet-50 encoder (Extended Data Fig. 2) aligns with these statistical results.

As expected, using a pathology foundation model improved the spatial quantification performance of all methods across all datasets (Supplementary Tables 3–7); however, SMMILe still demonstrates a notable advantage over these comparison methods (Fig. 4e,f). In most cases, the foundation model encoder resulted in spatial AUC and F1 score improvements of up to 30% (Fig. 4g,h). Because SMMILe had already achieved excellent results with the ImageNet-pretrained encoder, with spatial AUCs close to or exceeding 90%, its performance gains were smaller than those of other methods. Despite this, SMMILe still outperforms the top-4 methods in each dataset, achieving 3–6% higher spatial AUC scores on most datasets (Fig. 4i). While the foundation model markedly enhances the spatial quantification performance of these comparison methods—demonstrating good ranking capability between positive and negative patches (AUC score)—WSI-specific optimal thresholds are required for accurate spatial quantification. Therefore, AUC alone is insufficient to determine the real spatial quantification performance. In this regard, SMMILe not only achieves the highest spatial AUC scores but also learns more consistent classification boundaries within patch embedding spaces, outperforming the top-4 methods by margins of 5–20% in spatial F1 scores and 7–20% in spatial accuracy across most datasets (Fig. 4j and Supplementary Table 7). Notably, this advantage becomes more pronounced in challenging multilabel tasks, with SMMILe surpassing the top-4 methods by 5–10% in spatial F1 scores for Lung, Renal-3 and Renal-4, while achieving a larger margin of 7–20% for Gastric, Prostate and Gastric Endoscopy datasets.

The two exceptions are the Breast and Ovarian datasets. For the well-studied Breast metastasis detection dataset (Camelyon), a key characteristic is the small proportion of discriminative regions, as tumor areas typically comprise only a few patches in most metastasis cases. As a result, the issue of missed detection is diminished by the characteristics of this dataset. In addition, nearly all methods (except for TransMIL and AddMIL) achieved low false positive rates with the support of the pathology foundation encoder (Supplementary Table 4). Consequently, methods such as DSMIL, RAMIL and CLAM achieved spatial quantification performance comparable to SMMILe. For the partially annotated Ovarian dataset, the spatial quantification metrics are biased due to the limited number of annotated regions. Although SMMILe outperforms DTFD-MIL by only 1.14% in spatial AUC score, it achieves a much larger advantage in spatial accuracy, with a difference of 8.18%. Also, the spatial F1 score of SMMILe is 2.52% higher than CLAM, while its spatial accuracy surpasses CLAM by 6.29% (Supplementary Table 7).

Moreover, considering precision and recall, two additional spatial quantification metrics (Supplementary Tables 4 and 5), we observe that, while the pathology foundation model enhances spatial precision across various methods to a level comparable with SMMILe, it does not yield similar improvements in spatial recall. The baseline IAMIL achieves slightly lower false positive rates than SMMILe on several datasets (Lung, RCC-3 and RCC-4), while other methods (RAMIL, CLAM, DTFD-MIL, NIC and NICWSS) also demonstrate highly competitive spatial precision when integrated with the foundation model. However, despite foundation model integration, these methods struggle to mitigate missed detection issues, consistently falling behind SMMILe in spatial recall. For instance, SMMILe outperforms CLAM and DTFD-MIL by 5–15% and 9–40%, respectively, across all datasets in spatial recall. A notable exception is DSMIL, which achieves high spatial precision on some datasets (Breast and Ovarian) but suffers from suboptimal recall. Conversely, on other datasets (Lung and Renal-3), DSMIL exhibits high recall but low precision. This trade-off leads to its spatial F1 scores that are 6–28% lower than SMMILe, highlighting the challenge of simultaneously improving both recall and precision—even when using pathology foundation encoders.

**Fig. 5:**
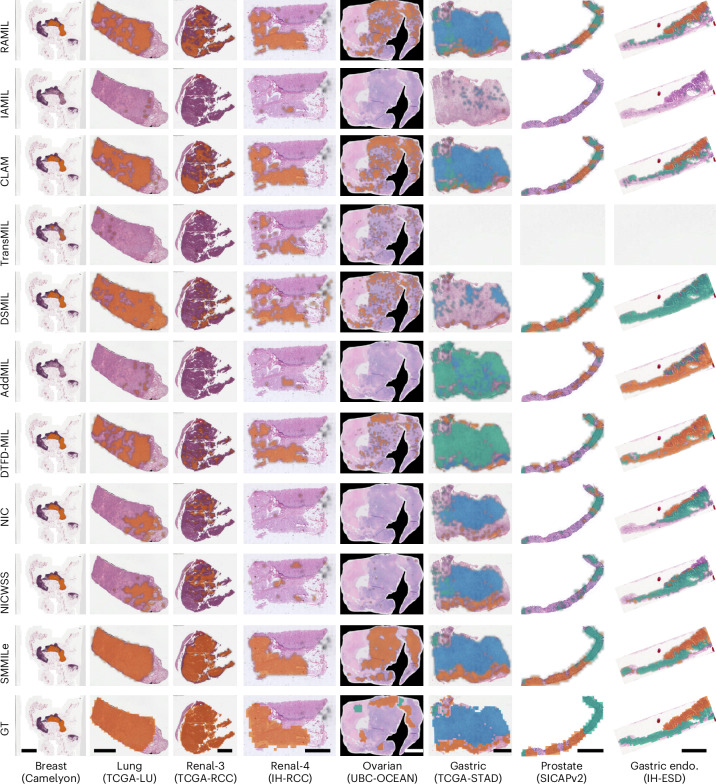
Visualization of spatial quantification across eight datasets using the pathology foundation model (Conch) as the encoder. For all datasets, to ensure uniform display, the GTs are shown as patch-level masks, while the spatial quantification results are displayed as patch-level masks with Gaussian smoothing applied. For the Breast, Lung, Renal-3, Renal-4 and Ovarian cases, orange represents tumor regions, while green in the Ovarian GT indicates normal tissue. In Gastric, Prostate and Gastric Endo. cases, different colors indicate different histotypes. In Gastric, orange, green and blue indicate poor differentiation, high differentiation and mucinous subtypes, respectively. In Gastric Endo., orange and green denote tumor and inflammation regions, respectively. In Prostate, G3 to G5 tumor regions are shown in orange, green and blue, respectively. Uncolored regions are classified or annotated as normal. Scale bar, 4 mm (applies to all columns).

Furthermore, the spatial visualization results (Fig. 5) also reveal that, even when equipped with the pathology foundation encoder and achieving strong overall metrics, these comparison methods still exhibit substantial false positive and false negative errors in several cases. In comparison, SMMILe exhibits superior spatial quantification performance, producing results nearly indistinguishable from the GT, even in challenging multilabel datasets.

The above results confirm that SMMILe successfully meets both objectives of precise WSI classification and spatial quantification on various datasets, achieving superior performance with both ImageNet pretrained and pathology-specific foundation encoders.

### Module integration improves spatial quantification performance

**Fig. 6:**
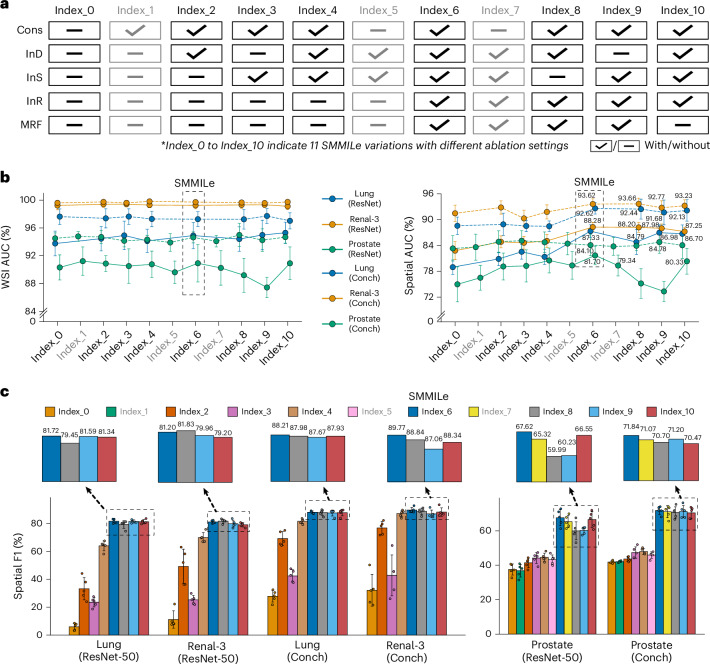
Ablation studies of SMMILe. **a**, Eleven ablation settings for SMMILe variations (index_0 to index_10). Dash and check marks indicate the absence and presence of each module, respectively. Settings related to Cons (index_1, index_5 and index_7) are shown in gray, indicating their application exclusively to specific datasets containing the normal category, specifically Prostate. **b**,**c**, Ablation results of SMMILe with ResNet-50 and Conch encoders on three representative datasets: Lung (*n* = 950 WSIs; 316,146 patches), Renal-3 (*n* = 660 WSIs; 290,061 patches) and Prostate (*n* = 153 WSIs; 18,426 patches). Data are presented as mean ± standard deviation (s.d.); error bars represent s.d. across fivefold cross-validation on independent tissue samples. In **b**, macro AUC scores for WSI classification and spatial quantification are shown from left to right. In **c**, macro F1 scores are reported for spatial quantification. Source data

We conducted comprehensive ablation studies (Fig. 6a) on three representative datasets—Lung, Renal-3 and Prostate—to assess the contribution of each module in SMMILe (Fig. 3a), including the consistency constraint (applied only to datasets with normal cases, such as Breast and Prostate), the parameter-free instance dropout (Fig. 3c), the delocalized instance sampling (Fig. 3d), the instance refinement network and the MRF constraint (Fig. 3e); each denoted as Cons, InD, InS, InR and MRF, respectively.

From the WSI classification results (Fig. 6b, left line chart), it is evident that the baseline SMMILe configuration without any modules (index_0) establishes a robust benchmark, attributable to the integration of the NIC feature compression layer with IAMIL. Integrating Cons markedly improves WSI classification performance for datasets with normal (negative) cases, as demonstrated by comparisons of index_0 versus index_1, index_4 versus index_5 and index_6 versus index_7 on the Prostate dataset, with consistent findings across these comparisons. Other modules have a minimal impact on WSI classification performance, particularly for the Lung and Renal-3 datasets when patch embeddings are extracted using the pathology foundation model.

From the patch-level spatial quantification results (Fig. 6b, the right line chart, and Fig. 6c), it is apparent that every module is essential for SMMILe with both ResNet-50 and Conch encoder. Integrating InD (index_2) or InS (index_3) individually, or both of them (index_4), brings notable improvements, but their effects vary across different datasets. For the Prostate dataset, both contribute substantially to the enhancement of spatial AUC and F1 scores. On the Renal-3 and Lung datasets, integrating InD (index_2) leads to a greater improvement in spatial F1 compared with integrating InS (index_3), while combining both (index_4) results in further improvement in spatial F1. However, in terms of spatial AUC, InS (index_3) shows a larger improvement on the Lung dataset (ResNet-50) compared with InD (index_2), whereas they show no noticeable difference in improvement on the Renal-3 (ResNet-50) dataset. The InR module (from index_5 to index_10) has a considerable impact across all three datasets; for instance, it results in a spatial AUC score improvement of 3–6% with the ResNet-50 encoder and 1–5% with the Conch encoder for the Lung and Renal-3 datasets. Although InR does not show substantial improvement on the Prostate dataset in terms of spatial AUC score due to the class imbalance issue among patches, it substantially boosts the spatial F1 score by more than 20%, far exceeding the gains observed in the other two datasets.

Furthermore, the results from index_6 to index_10 reflect the impact of removing each module on the overall spatial quantification performance. It is evident that, for the Lung and Renal-3 datasets, the influence of each module on spatial AUC is relatively minor, whereas their impact on spatial F1 is more pronounced. Specifically, by leveraging spatial smoothness (index_6 versus index_10), MRF enhances the spatial F1 score by 0.4% to 2%. Also, removing InS (index_8) or InD (index_9) has varying effects across different settings and datasets. With the ResNet-50 encoder, removing InS (index_8) led to a more than 2% decrease in the spatial F1 score on the Lung dataset, while it resulted in a 1.6% improvement on the Renal-3 dataset. Conversely, removing InD (index_9) resulted in only a 0.1% drop in the spatial F1 score on the Lung dataset but caused a 1.2% decrease on the Renal-3 dataset. When using the Conch encoder, removing either module led to a performance decline, with InD (index_9) having a greater impact, causing around a 3% drop on the Renal-3 dataset. For the more challenging Prostate dataset, removing any module substantially impacts spatial quantification performance. Specifically, removing either InS (index_8) or InD (index_9) with the ResNet-50 encoder results in a decrease of approximately 17% in the spatial F1 score and 6–8% in the spatial AUC score. Even with the Conch encoder, the spatial F1 score drops by 0.6–2%. Moreover, Cons also plays a positive role in spatial quantification, with its removal (index_7) leading to an approximate 2% drop in the spatial F1 score.

The comprehensive ablation experiments demonstrate the crucial role of each module, particularly in spatial quantification. SMMILe is a holistic MIL method, where integrating all modules is essential for it to achieve consistently superior performance across different settings and datasets. We also investigate the effect of applying each module to RAMIL (see Supplementary Table 8 and ‘Cross-model ablation’ in the Supplementary Note for details).

### Impact of annotation precision on spatial quantification

**Fig. 7:**
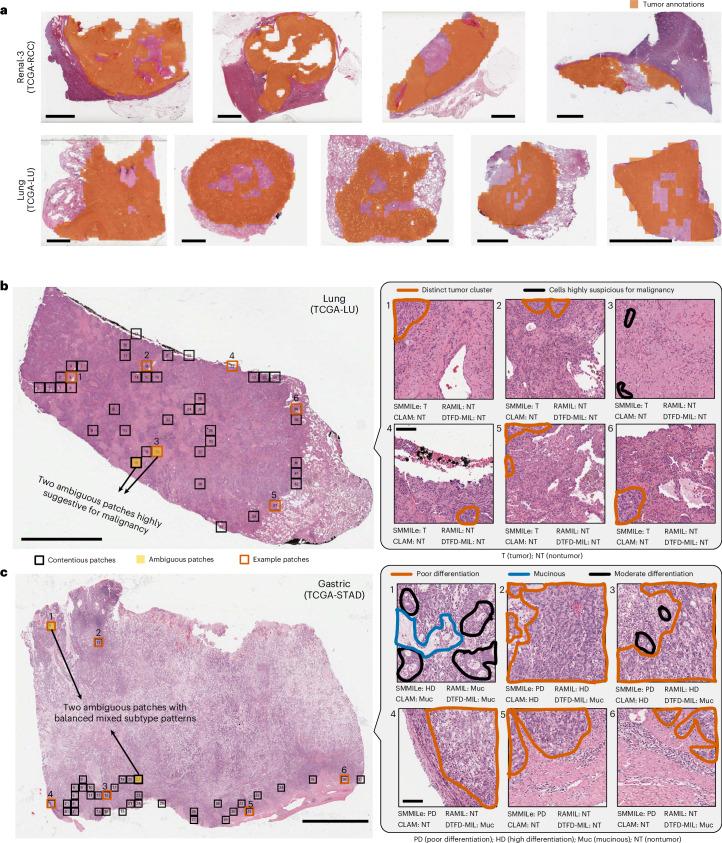
Case studies of spatial quantification using the pathology foundation model (Conch) as the encoder. **a**, Patch-level GTs from the Renal-3 and Lung datasets, with orange indicating tumor regions, ensuring the exclusion of large nontumor areas within each tumor region. **b**, The case from the Lung dataset. Orange contours on the patches indicate tumor areas, while black denotes highly suspected undifferentiated tumor cells. **c**, The case from the Gastric dataset. Orange and blue contours on the patches indicate poor differentiation and mucinous areas, respectively, while black denotes moderate differentiation without reaching high differentiation. The boxes in the WSIs indicate contentious patches, with yellow-shaded patches indicating those were reviewed and found to have ambiguous labels. Six patches per case from different regions are magnified and visualized in high resolution with the diagnostically relevant areas outlined. Scale bars, 4 mm (for all WSIs in **a**–**c**) and 80 µm (for the corresponding patches in **b** and **c**).

The assessment of spatial quantification is inherently influenced by annotation precision on the hematoxylin and eosin (H&E) slides. In tumor region annotations, small stroma areas within tumors may not be fully excluded. Despite our efforts to ensure precise annotation (Fig. 7a), some nontumor tissue may still be included.

To investigate further the potential effects of annotation precision on spatial quantification, we analyzed two representative cases. We defined contentious patches that may impact spatial quantification evaluation as those where SMMILe’s predictions align with the GT, while all three runner-up models (RAMIL, CLAM and DTFD-MIL) fail to do so. An experienced pathologist reviewed all contentious patches to assess annotation accuracy and identify potential sources of discrepancy.

In the Lung cancer case (Fig. 7b), most contentious patches are located at tumor margins or intratumor stroma regions, with only 2 out of 42 patches having ambiguous labels, both of which contain cells highly suspicious for undifferentiated malignancy. Among the high-resolution example patches, all except for patch 3 (patches 1, 2 and 4–6) can be regarded as low-discriminative positive instances, where some distinct tumor cell clusters are present. The original GTs are correct, and SMMILe successfully identifies these patches.

In the Gastric cancer case (Fig. 7c), contentious patches appear at both tumor margins and subtype boundaries, with only 2 out of 37 patches having ambiguous labels, both of which contain mixed subtype patterns. Among the high-resolution patches, most patches (2–6) have accurate GTs and are correctly classified by SMMILe, while one patch with an ambiguous label appears in a subtype transitional zone. Patch 1, annotated as highly differentiated, contains a clear mucinous pattern, suggesting it could be more accurately reannotated as mucinous.

Overall, this analysis suggests that, although annotation precision affects spatial quantification in this study, its overall impact remains minimal.

## Discussion

A vast number of weakly supervised learning approaches, particularly MIL methods, have been developed for WSI analysis using readily available patient-level diagnostic labels. Most of them utilize attention-based representation-level aggregation for precise WSI classification but overlook the spatial quantification required for many critical tasks and discoveries in clinical pathology. Although these representation-based MIL methods can offer spatial interpretability through the visualization of attention scores, which seems capable of achieving spatial quantification, few works quantitatively assess their interpretability. Here, we conducted a detailed evaluation of WSI-level classification and spatial quantification on 9 state-of-the-art (SOTA) weakly supervised WSI classification methods across eight diverse datasets and six cancer types. In addition to common binary and multiclass classification datasets, we also assessed three more complex multilabel classification datasets. We demonstrated that, although existing methods can achieve satisfactory WSI classification performance, their spatial quantification capabilities are inadequate for clinical applications and downstream analysis, even when integrated with powerful pathology foundation models.

In this study, we studied the theoretical underpinnings of how attention scores are allocated differently across MIL methodologies, specifically between RAMIL and IAMIL. These findings were further corroborated by synthetic experimentation. Theoretical exploration and synthetic experimental evidence suggest that, despite its inherent challenges—such as relatively inferior bag-level prediction capabilities and high precision but low recall in positive instance detection—IAMIL holds superior potential for spatial quantification and interpretability in WSI analysis compared with RAMIL. Subsequently, we illustrated that, by integrating NIC, comprehensive attention modules and an instance refinement network into the IAMIL framework, it is possible to develop a robust MIL method, SMMILe. Compared with other SOTA MIL methods, SMMILe not only demonstrates superior and consistent WSI classification performance but also delivers excellent spatial quantification across a wide range of pathology tasks—including metastasis detection, subtyping and grading—using only patient-level labels for model training.

Furthermore, we demonstrated that the advancements in performance can be directly linked to the innovative modules integrated within SMMILe. Specifically, NIC enhances SMMILe by enabling convolution operations that expand the local receptive field of instance embeddings, thereby improving WSI classification performance. Two advanced comprehensive attention modules (parameter-free instance dropout and delocalized instance sampling) elevate SMMILe’s ability to recognize positive patches with relatively low-discriminative features, improving its ability to differentiate between such patches and those that are negative. Moreover, as both modules incorporate stochastic processes that generate unique instance arrangements in each iteration—resembling WSI-level data augmentation—they further contribute to enhancing the classification performance and robustness of SMMILe at the WSI level. Building on these enhancements, the instance refinement network addresses a prevalent challenge in attention-based MIL methods: the variability in the optimal decision threshold for attention scores at the patch level across different WSIs. This variability complicates the task of selecting a universal threshold. The network markedly bolsters SMMILe’s ability to discriminate at the patch level by learning a unified decision boundary across different WSIs. When combined with the MRF energy constraint, it further improves the spatial coherence of patch-level predictions, ensuring smoother transitions and consistency in classifications across contiguous patches.

This work has certain limitations. While the evaluation has been performed on eight diverse datasets, it remains focused on traditional computational pathology tasks owing to dataset availability. However, spatial quantification in more advanced challenges may provide even more added value. For example, it may be possible to generate spatially accurate predictions of immunohistochemical or genomic biomarkers from H&E; these predictions could be validated with the corresponding immunohistochemical images or spatially resolved sequencing data.

Moreover, spatial quantification is influenced by patch size and the criteria for defining patch labels used in this study. Specifically, in binary and multiclass classification, a patch is labeled as tumor if it contains any tumor cells, whereas in multilabel classification, the label is assigned based on the dominant histological category. These criteria require further standardization to ensure consistency across studies.

In conclusion, a spatial quantification framework like SMMILe possesses great potential to be applied in multiple research and clinical contexts. For general pathology diagnostic tasks, SMMILe not only provides slide-level diagnoses but also offers a reliable visualization to support explainability. For tasks involving the quantitative analysis of pathology phenotypes, SMMILe enables precise spatial quantification, further supporting extensive retrospective studies aimed at exploring the relationship between the proportions of different pathological phenotypes and patient treatment responses or prognostic evaluations. For the task of discovering biomarkers, SMMILe has the potential to identify and quantify previously unknown pathological phenotypes, thereby facilitating subsequent targeted and quantitative analyses at the genomic level, such as spatial sequencing of subregions.

## Methods

### Data collection and processing

Most of the datasets analyzed in this work were collected from publicly released research resources and databases. For the in-house datasets (IH-RCC and IH-ESD), data collection was conducted with approval from the Ethics Committee of the First Affiliated Hospital of Xi’an Jiaotong University (approval numbers KYLLSL2021-420 and KYLLSL2022-333). The committee also approved a waiver of informed consent. All patient data were fully anonymized to protect privacy and did not contain any personally identifiable information or protected health details. No participant compensation was involved. This study involved only WSI analysis without collecting or using sex- or gender-related information. The eight histopathology datasets are listed as follows, all of which are derived from formalin-fixed, paraffin-embedded tissue sections.

The Breast (Camelyon16)^33^ dataset contains 399 WSIs with pixel-level metastasis annotations. Lung (TCGA-LU, 937 WSIs) includes two subtypes, adenocarcinoma (LUAD)^34^ and squamous cell carcinoma (LUSC)^35^, with 523 WSIs having pixel-level cancer annotations. Ovarian (UBC-OCEAN, 513 WSIs)^40^ comprises five subtypes; 152 WSIs were partially annotated as healthy/cancerous/necrotic, with health/necrotic regions categorized as normal. RCC-3 (TCGA-RCC, 660 WSIs) includes three RCC subtypes, clear cell (CCRCC)^36^, papillary (PRCC)^37^ and chromophobe (CHRCC)^38^, with 338 WSIs having full pixel-level cancer annotations. RCC-4 (IH-RCC, 563 WSIs) includes four RCC subtypes with 138 annotated WSIs. Gastric Endoscopy (IH-ESD, 99 WSIs from 286 samples) was annotated for tumor and inflammation. Gastric (TCGA-STAD, 339 WSIs)^41^ was subtype-labeled by expert pathologists, and 128 WSIs received patch-level annotations. Prostate (SICAPv2, 153 WSIs)^42^ includes Gleason grades G3–G5 and normal tissue, with missing annotations added by expert pathologists. See Table 1 and and ‘Dataset description’ in the Supplementary Note for more details.

Following the CLAM^43^ preprocessing pipeline, we tessellated WSIs into nonoverlapping tiles: 2,048 × 2,048 at 40× and 1,024 × 1,024 at 20× for TCGA datasets, and 512 × 512 at 20× for Ovarian and Gastric Endoscopy. Breast WSIs were tiled at 512 × 512 at 40× due to small tumor regions. Prostate used existing 512 × 512 tiles with 50% overlap. Patch labels were assigned based on the dominant pixel-level annotation. WSIs labeled as ‘Normal’ contained uniformly negative instances.

### RAMIL versus IAMIL

We conduct a theoretical investigation into the mechanistic differences between RAMIL and IAMIL, formulating three original theorems and designing a targeted synthetic experiment to address two fundamental questions: (1) How does the variation in the stage of attention aggregation lead to noticeable differences in the attention score allocation? (2) How do these differences contribute to a reduced false positive rate in IAMIL compared with RAMIL?

Our exploration of these questions reveals that during training IAMIL tends to assign lower attention scores to low-discriminative or nondiscriminative instances and higher scores to highly discriminative ones, in contrast to the distribution patterns observed in RAMIL.

Furthermore, it also disproves some previous interpretations regarding the inaccurate attention results in RAMIL^28^. Contrary to claims attributing this issue to conflicting instance contributions or interactions at the classification stage, our findings suggest a different explanation. In RAMIL, bag-level prediction logits can indeed be precisely decomposed into marginal instance contributions as determined by the instance attention scores. However, the properties of the output activation function result in a diminished contribution of these attention scores to the bag-level prediction score. Consequently, during optimization, the loss constraint provides limited guidance for attention score allocation, resulting in some negative instances receiving relatively high attention scores.

### Preliminaries

Let $\{{{\bf{x}}}_{1},{{\bf{x}}}_{2},\ldots ,{{\bf{x}}}_{K}\}$ be a bag of instances (patches) extracted from a WSI ${\bf{X}}\in {{\mathbb{R}}}^{W\times H\times 3}$, with WSI-level label ${\bf{Y}}$ as supervision, where each patch ${{\bf{x}}}_{k}\in {{\mathbb{R}}}^{D\times D\times 3}$ and $K$ represents the number of instances. In WSI analysis, representation-based MIL approaches dominate due to their superior bag-level prediction performance^49–52^, and typically involve three essential steps, which are as follows: (1) Instance embedding via the pretrained encoder $e(\cdot )$, yielding {**h**_**1**_, **h**_**2**_,…,**h**_**K**_}, where ${{\bf{h}}}_{{\bf{k}}}=e({{\bf{x}}}_{k})$. (2) Aggregation into a bag-level representation ${\bf{H}}$ by a permutation-invariant function (for example, mean and max pooling). (3) Mapping ${\bf{H}}$ to category prediction scores ${\bf{P}}=\{{P}_{1},{P}_{2},\ldots ,{P}_{C}\}$ using the linear projection function $f(\cdot )$ with an output activation function $\sigma (\cdot )$ (for example, sigmoid and softmax), where $C$ stands for the number of categories (see Supplementary Table 9 for detailed description of key variables). Taking mean pooling as an example, the bag-level prediction score ${\bf{P}}$ is computed as

1 $${\bf{P}}=\sigma \left(f\left(\frac{1}{K}\mathop{\sum }\limits_{k=1}^{K}{{\bf{h}}}_{k}\right)\right).$$

However, this framework struggles to assign interpretable scores to individual instances, which is crucial in medical applications^53^. Attention-based MIL^32^ (RAMIL) addresses this by introducing a learnable attention mechanism over instance representations with an auxiliary network $g(\cdot )$, providing instance-level interpretability through attention weights $\{{a}_{1},{a}_{2},\ldots ,{a}_{K}\}$. Consequently, the bag-level prediction score ${\bf{P}}$ of RAMIL can be reformulated as

2 $${\bf{P}}=\sigma \left(f\left(\mathop{\sum }\limits_{k=1}^{K}{a}_{k}\times {{\bf{h}}}_{k}\right)\right).$$

In contrast to RAMIL, whose visual interpretations have been shown to be inexact and incomplete^26,28^, instance-based MIL (IAMIL) computes prediction scores at the instance level before aggregation, enabling more direct and reliable interpretability. The bag-level prediction score ${\bf{P}}$ with the mean pooling can be formulated as

3 $${\bf{P}}=\frac{1}{K}\mathop{\sum }\limits_{k=1}^{K}\sigma \left(\,f({{\bf{h}}}_{k})\right).$$

Instance-based MIL typically treats the bag label as a pseudo-label for all instances, leading to inaccurate supervision, especially for nondiscriminative instances. This weak supervision limits the effectiveness of the projection function $f(\cdot )$ in equation (3), ultimately affecting the bag-level prediction^54^. To address this issue, an attention mechanism can be introduced directly over instance scores to reweight their contributions and suppress noisy signals. The resulting bag-level prediction score ${\bf{P}}$ in IAMIL can be calculated as

4 $${\bf{P}}=\mathop{\sum }\limits_{k=1}^{K}{a}_{k}\times \sigma \left(\,f({{\bf{h}}}_{k})\right).$$

This formulation aligns with the multiple-instance detection network (MIDN)^31^ used in weakly supervised objective detection (WSOD) where images are bags and object proposals are instances. From equation (2) and equation (4), the key difference between RAMIL and IAMIL lies in the stage where attention is applied (Fig. 2b). Considering observations from substantial existing related works^24–31^, and given that spatial quantification in both methodologies fundamentally relies on the attention scores ($A=\{{a}_{1},{a}_{2},\ldots ,{a}_{K}\}$), it is reasonable to assume that even a slight distinction between them may lead to notable variation in attention score allocation. This insight prompts us to explore how these two methodologies diverge in their mechanisms for assigning instance-level attention within this foundational MIL framework.

### Connecting RAMIL with IAMIL

We start by converting the formulation of RAMIL (equation (2)) into a form that parallels with IAMIL.

#### Theorem 1

*RAMIL is rigorously equivalent to a specialized variant of IAMIL, wherein aggregation is conducted at the logit level,*

5 $$\sigma \left(f\left(\mathop{\sum }\limits_{k=1}^{K}{a}_{k}\times {{\bf{h}}}_{k}\right)\right)=\sigma \left(\mathop{\sum }\limits_{k=1}^{K}{a}_{k}\times f({{\bf{h}}}_{k})\right).$$

This specific form of MIL termed logits-based attention MIL (LAMIL) was initially utilized in WSOD settings^55^, although without attention pooling (see ‘Proof of theorem 1’ in the Supplementary Note).

### The gradient descent process of attention MIL

Building upon theorem 1, the core divergence between RAMIL and IAMIL lies in where the activation function $\sigma (\cdot)$ is applied (equation (4) versus the right side of equation (5)). For clarity in the subsequent derivation, we generalize both of them by incorporating class-wise attention and adopting the sigmoid function as $\sigma (\cdot)$. Consequently, the attention score ${a}_{k}$ is expanded to a vector ${{\bf{a}}}_{k}=\{{a}_{k}^{1},{a}_{k}^{2},\ldots ,{a}_{k}^{C}\}$, where ${a}_{k}^{c}$ denotes the softmax normalized attention score for the $k$th instance of category $c$. This formulation extends attention-based MIL to multilabel tasks.

Moreover, as the softmax function introduces interdependencies among attention scores across instances, we explicitly represent the normalized attention score as ${a}_{k}^{c}={{\rm{e}}}^{{z}_{k}^{c}}/\sum_{j=1}^{K}{{\rm{e}}}^{z_{j}^{c}}$ and analyze the gradient descent process of ${z}_{k}^{c}$ in RAMIL and IAMIL. Let ${{\bf{l}}}_{k}=\{{l}_{k}^{1},{l}_{k}^{2},\ldots ,{l}_{k}^{C}\}$ represent the logits from the linear projection function $f(\cdot )$, where ${{\bf{l}}}_{k}=f({{\bf{h}}}_{k})$ and each ${l}_{k}^{c}$ corresponds to category $c$. The aggregated logit for category $c$, denoted as ${L}_{c}$, is defined as

6 $${L}_{c}=\mathop{\sum }\limits_{k=1}^{K}\frac{{{\rm{e}}}^{{z}_{k}^{c}}}{\sum_{j=1}^{K}{{\rm{e}}}^{z_{j}^{c}}} {l}_{k}^{c}.$$

Accordingly, the predicted score for category $c$ in RAMIL is calculated as follows:

7 $$\check{{P}_{c}}=\sigma (\check{{L}_{c}})=\frac{1}{1+{{\rm{e}}}^{-{\sum }_{k=1}^{K}\frac{{{\rm{e}}}^{\check{{z}_{k}^{\,c}}}}{\sum_{j=1}^{K}{{\rm{e}}}^{\check{z_{j}^{\,c}}}} \check{{l}_{k}^{c}}}},$$

and the prediction score for category $c$ in IAMIL is given by

8 $${\hat{P}_{c}}=\mathop{\sum }\limits_{k=1}^{K}{\hat{{a}_{k}^{c}}} {\hat{{p}_{k}^{c}}}=\mathop{\sum }\limits_{k=1}^{K}\frac{{{\rm{e}}}^{{\hat{{z}_{k}^{c}}}}}{\sum_{j=1}^{K}{{\rm{e}}}^{{\hat{{z}_{\!j}^{c}}}}} \frac{1}{1+{{\rm{e}}}^{-{\hat{{l}_{k}^{c}}}}},$$

where ${\hat{{p}_{k}^{c}}}=\sigma ({\hat{{l}_{k}^{c}}})$. To theoretically explore the variation in the attention score allocation under conditions where RAMIL and IAMIL are optimized to the same level, we posit the following assumptions:

#### Assumption 1

Except for the two attention networks, denoted as ${\check{g}}(\cdot)$ and $\hat{g}(\cdot )$, both models share identical parameters in their pretrained encoders $e(\cdot )$ and linear projection functions $f(\cdot )$, which means ${\hat{{l}_{i}^{c}}}={\check{{l}_{i}^{c}}}$ for any instance ${{\bf{x}}}_{i}$ and category $c$.

#### Assumption 2

Upon training with the same dataset and using an identical loss function, $\exists$ $\check{{g}_{c}}(\cdot )$, $\hat{{g}_{c}}(\cdot )$ with weight $\check{{{\bf{w}}}_{g}^{c}}$, $\hat{{{\bf{w}}}_{g}^{c}}$, for any $c\in [1,C]$ and $k\in [1,K-1]$, the following two conditions are satisfied: (1) the bag-level predictions of RAMIL and IAMIL for the same inputs are identical, that is, $\sigma ({\check{L}_{c}})={\check{P}_{c}}={\hat{P}_{c}}$, for any category $c$. (2) The gradients of the final losses with respect to the respective network parameters are equivalent, that is, $\frac{\partial \check{{{\mathscr{L}}}_{c}}}{\partial \check{{{\bf{w}}}_{g}^{c}}}=\frac{\partial \hat{{{\mathscr{L}}}_{c}}}{\partial \hat{{{\bf{w}}}_{g}^{c}}}$.

#### Theorem 2

*If assumptions 1 and 2 hold, the relationship in magnitude between* $\check{{a}_{i}^{c}}$ *and* $\hat{{a}_{i}^{c}}$ *is directly proportional to the corresponding magnitude relationship between* $(\sigma ({L}_{c})-\sigma ({l}_{i}^{c}))$ *and* $\frac{\partial \sigma ({L}_{c})}{\partial {L}_{c}}({L}_{c}-{l}_{i}^{c})$, *which is valid for any instance* ${{\bf{x}}}_{i},i\in \{1,2,\ldots ,K-1\}$:

9 $$\frac{\check{{a}_{i}^{c}}}{\hat{{a}_{i}^{c}}}=\frac{\sigma ({L}_{c})-\sigma ({l}_{i}^{c})}{\frac{\partial \sigma ({L}_{c})}{\partial {L}_{c}}({L}_{c}-{l}_{i}^{c})}.$$

Theorem 2 addresses the first question, asserting that the difference in the attention score allocation between RAMIL and IAMIL is intrinsically linked to the properties of the activation function $\sigma (\cdot )$ across the relevant interval (see ‘Proof of theorem 2’ in Supplementary Note).

### Properties of activation function in local intervals

Based on theorem 2, we can infer that if the absolute distance $|\sigma ({L}_{c})-\sigma ({l}_{i}^{c})|$ surpasses the projection of $|{L}_{c}-{l}_{i}^{c}|$ on the tangent line $t(l)$, then the attention score $\check{{a}_{i}^{c}}$ allocated to the *i*th instance by RAMIL exceeds that assigned by IAMIL $\hat{{a}_{i}^{c}}$, where $t(l)$ denotes the tangent line to $\sigma (l)$ at ${L}_{c}$. Conversely, if the projection on the tangent line is greater, the result is reversed.

Also, the activation function (sigmoid) exhibits concavity in the interval [0, ∞) and convexity in the interval (−∞, 0]. This dichotomous property markedly affects the relative magnitudes of $\check{{a}_{i}^{c}}$ and $\hat{{a}_{i}^{c}}$. As a result, the magnitude relationship between them is not fixed but is intricately linked to the interval demarcated by ${L}_{c}$ and ${l}_{i}^{c}$.

#### Theorem 3

*If* $|{l}_{i}^{c}| > |{L}_{c}|$ *and* ${L}_{c}\cdot {l}_{i}^{c} > 0$, *then* $\check{{a}_{i}^{c}} < \hat{{a}_{i}^{c}}$. *Conversely, if* ${l}_{i}^{c}\in (\min ({L}_{c},{L}_{\mathrm{int}}),\max ({L}_{c},{L}_{\mathrm{int}}))$*, then* $\check{{a}_{i}^{c}} > \hat{{a}_{i}^{c}}$. *where* ${L}_{\mathrm{int}}$ *is the additional intersection point between* $t(l)$ *and* $\sigma (l)$.

Typically, the *i*th instance is considered to possess high discriminability if $|{l}_{i}^{c}| > |{L}_{c}|$ and ${L}_{c}\cdot {l}_{i}^{c} > 0$. By contrast, the instances are regarded as relatively low-discriminative or nondiscriminative instances. Furthermore, owing to the specific properties of the additional intersection point, the conditions outlined in theorem 3 cover nearly all instances, and few instances are likely to satisfy the rest condition, that is, $|{l}_{i}^{c}| > |{L}_{\mathrm{int}}|$ and ${L}_{\mathrm{int}}\cdot {l}_{i}^{c} > 0$ (see ‘Proof of theorem 3’ in Supplementary Note).

Theorem 3 responds to the second question, highlighting that IAMIL, in contrast to RAMIL, tends to give higher attention scores to high-discriminative instances. Simultaneously, it assigns markedly lower attention scores—often several times lower—to instances with relatively low discriminability or those that are nondiscriminative. This results in a substantially lower false positive rate compared with RAMIL, but also leads to reduced recall, as only a limited subset of truly discriminative instances receives sufficiently high attention scores.

The aforementioned statements are also validated in a synthetic experiment, see ‘Synthetic experiment’ in the Supplementary Note for details.

### SMMILe

Reiterating the definition in ‘Preliminaries’, a set of patches $\{{{\bf{x}}}_{1},{{\bf{x}}}_{2},\ldots ,{{\bf{x}}}_{K}\}$, each of size $D\times D$, are extracted from a WSI ${\bf{X}}$ of size $W\times H$, with the corresponding label $Y$. The objective of SMMILe is to learn a transformation function that maps the set of patches $\{{{\bf{x}}}_{1},{{\bf{x}}}_{2},\ldots ,{{\bf{x}}}_{K}\}$ to the WSI-level label ${\bf{Y}}$. Concurrently, it also aims to predict instance-level labels ${\{y}_{1},{y}_{2},\ldots ,{y}_{K}\}$ for each individual patch.

In MIL settings, supervision is exclusively available at the WSI level. Previous research has predominantly focused on binary or multiclass classification, where each WSI is assigned to a single category, that is, ${\bf{Y}}$ is represented as a scalar in binary classification or as a $C$-dimensional one-hot vector in multiclass classification. In this Report, we expand SMMILe to accommodate multilabel classification. Consequently, ${\bf{Y}}=\{{Y}_{1},{Y}_{2},\ldots ,{Y}_{C}\}$ is configured as a $C$-dimensional vector, with each element ${Y}_{c}$ being a binary indicator that is independently distributed, representing the presence or absence of each category in this WSI. This adaption aligns SMMILe with more general pathology scenarios, capturing multiple phenotypic categories that may concurrently exist in a single WSI.

### Network architecture

Our architecture (Fig. 3a,b) begins with a pretrained encoder $e(\cdot )$. Feature maps are extracted per patch. Given the large number of instances (patches) per WSI, the encoder remains frozen for computational efficiency. A trainable convolutional layer $\mathrm{cov}(\cdot )$ is then applied to map embeddings to a lower dimension. Unlike standard linear projections, we use 3 × 3 kernels over spatially rearranged instance embeddings (following NIC^45^), enabling local context modeling.

Each compressed embedding is processed by an instance detector $g(\cdot )$ and classifier $f(\cdot )$. The detector, using a gated attention mechanism^32^, outputs raw attention scores per category, normalized via softmax across instances. The classifier maps each instance to per-category logits, normalized via softmax (multiclass) or sigmoid (binary/multilabel). Final WSI-level predictions are computed as the dot product of attention and classification scores across instances (see equation (4)).

### Instance-based comprehensive attention

To better capture all discriminative instances, SMMILe incorporates three key components, treating WSIs as either positive bags (containing at least one positive instance) or negative bags (no positive instances). These components include (1) an attention consistency constraint for negative bags; (2) a parameter-free instance dropout module; and (3) a superpatches-based delocalized instance sampling module for positive bags.

#### Consistency constraint

In contrast to previous RAMIL approaches, we apply different attention mechanisms to positive and negative bags. For negative bags, we enforce uniform attention across all instances using a mean squared error loss:

10 $${ {\mathcal L} }_{\mathrm{cons}}=\frac{1}{CK}\mathop{\sum }\limits_{c=1}^{C}\mathop{\sum }\limits_{k=1}^{K}{({a}_{k}^{c}-{\bar{a}}^{c})}^{2},$$

where ${\bar{a}}^{c}=\frac{1}{K}{\sum }_{k=1}^{K}{a}_{k}^{c}$. This discourages the model from focusing on specific patches, improving negative bag recognition and attention contrast.

#### Parameter-free instance dropout

MIL models often overfit to a few highly discriminative instances. To counter this, we introduce a dropout mechanism (Fig. 3c) that stochastically removes such instances during training, forcing the model to learn from other informative regions. Crucially, this dropout is parameter-free, adapting to each bag. Instead of dropping based on attention scores, we use the instance-level contributions ${I}_{k}^{c}={a}_{k}^{c} {p}_{k}^{c}$, which directly determines bag-level prediction. These scores are min–max normalized and compared against uniformly drawn random values ${\eta }_{k}^{c}\in \left[0,1\right]$ to generate dropout masks ${O}_{k}^{c}=\left[{\breve{I}}_{k}^{c} < {\eta }_{k}^{c}\right].$ The resulting bag-level prediction score is

11 $${P}_{c}^{\mathrm{dp}}=\mathop{\sum }\limits_{k=1}^{K}{O}_{k}^{c} {I}_{k}^{c}.$$

This strategy enhances robustness by encouraging the model to consider lower-confidence yet still relevant patches.

#### Superpatch-based delocalized instance sampling

To further promote comprehensive attention, we introduce a spatially aware sampling strategy (Fig. 3d). Instead of random instance subsets, we use superpatches, that is, spatial clusters of similar patches generated using simple linear iterative clustering (SLIC)^56^ of the compressed WSI (Fig. 3b). Each WSI is divided into $S$ superpatches, from which one instance is sampled per round to form a pseudo-bag, with the bag-level prediction score for the $t$th pseudo-bag being

12 $${P}_{c,t}^{\mathrm{sp}}=\mathop{\sum }\limits_{s=1}^{S}{\widetilde{I}}_{s,t}^{c}.$$

This ensures each pseudo-bag samples spatially and morphologically distinct regions, promoting broader coverage of tissue phenotypes. To prevent overreliance on dominant regions, we also apply instance dropout to these sampled bags:

13 $${P}_{c,t}^{\mathrm{sdp}}=\mathop{\sum }\limits_{s=1}^{S}{O}_{s}^{c} {\widetilde{I}}_{s,t}^{c}.$$

This combination is especially helpful in tumor subtyping tasks, where many instances may be positive and high-confidence patches risk overshadowing weaker signals.

Finally, all bag-level predictions—original, dropout, sampled and dropout sampled—are supervised by the bag-level label ${Y}_{c}$ of each category. The classification loss for each bag is calculated as

14 $$\begin{array}{l}{{\mathscr{L}}}_{\mathrm{cls}}=\frac{1}{C}\mathop{\sum }\limits_{c=1}^{C}\left(\text{BCE}({P}_{c},{Y}_{c})+\text{BCE}({P}_{c}^{\mathrm{dp}},{Y}_{c})\right)\\ +\frac{1}{{CT}}\mathop{\sum }\limits_{t=1}^{T}\mathop{\sum }\limits_{c=1}^{C}\left(\text{BCE}({P}_{c,t}^{\mathrm{sp}},{Y}_{c})+\text{BCE}({P}_{c,t}^{\mathrm{sdp}},{Y}_{c})\right)\end{array},$$

where $\text{BCE}(P,Y)$ stands for the binary cross-entropy loss between the prediction $P$ and the true label $Y$.

### MRF-based instance refinement

To enable consistent instance-level predictions across diverse WSIs, SMMILe introduces an instance refinement network (Fig. 3e) that aligns features of patches belonging to the same category across different bags. This addresses variability in feature distributions and positive-instance ratios across bags, which makes it difficult to set a universal decision boundary.

#### Instance refinement network

The refinement module consists of $N$ linear layers $\{{v}_{1}(\cdot ),{v}_{2}(\cdot ),\ldots ,{v}_{N}(\cdot )\}$, each outputting a $\left(C+1\right)$-dimensional softmax score, where $C$ is the number of classes. Each layer is trained with pseudo-labeled instances selected during training. Pseudo-labels are derived from SMMILe’s instance scores ${I}_{k}^{c}={a}_{k}^{c}\cdot {p}_{k}^{c}$. For a positive category $c$, the top $\theta$% of instance scores are selected. For the negative class, we take the bottom $\theta$% of mean instance scores across all categories, ${\bar{I}}_{k}=\frac{1}{C}{\sum }_{c=1}^{C}{I}_{k}^{c}$.

Each linear layer is trained using these pseudo-labeled instances. Subsequent layers use predictions from the previous layer as soft supervision, similar to a self-training scheme, allowing progressive refinement:

15 $${ {\mathcal L} }_{\mathrm{ref}}=\frac{1}{NJ}\mathop{\sum }\limits_{n=1}^{N}\mathop{\sum }\limits_{j=1}^{J}\mathrm{CE}({{\bf{p}}}_{j}^{n},{\breve{y}}_{j}^{n}),$$

where ${{\bf{p}}}_{j}^{n}$ is the predicted probability vector from layer ${v}_{n}$, ${\breve{y}}_{j}^{n}$ is the pseudo-label and $\text{CE}({\bf{p}},y)$ is the categorical cross-entropy loss.

This joint training improves consistency in instance features across bags and strengthens WSI-level predictions.

#### Superpatch-based MRF constraint

While pseudo-labeling captures category-specific features, it ignores spatial context. To address this, SMMILe introduces a superpatch-based MRF constraint (Fig. 3e) that encourages local spatial smoothness in predictions.

WSIs are divided into superpatches using SLIC. Within each superpatch ${\text{SP}}_{s}$, instance predictions $\{{{\bf{p}}}_{1}^{n},\ldots ,{{\bf{p}}}_{|{\text{SP}}_{s}|}^{n}\}$ are encouraged to be similar (first-order term). In addition, predictions between adjacent superpatches are smoothed (second-order term):

16 $${ {\mathcal L} }_{\mathrm{mrf}}=\frac{1}{N}\mathop{\sum }\limits_{n=1}^{N}\left(\frac{{\lambda }_{1}}{|{\mathrm{SP}}_{s}|}\mathop{\sum }\limits_{sp=1}^{|{\mathrm{SP}}_{s}|}{\Vert {{\bf{p}}}_{sp}^{n}-{\bar{{\bf{p}}}}_{s}^{n}\Vert }^{2}+\frac{{\lambda }_{2}}{{M}_{s}}\mathop{\sum }\limits_{m=1}^{{M}_{s}}{\Vert {\bar{{\bf{p}}}}_{s,m}^{n}-{\bar{{\bf{p}}}}_{s}^{n}\Vert }^{2}\right),$$

where ${\bar{{\bf{p}}}}_{s}^{n}$ is the mean prediction within a superpatch and ${\bar{{\bf{p}}}}_{s,m}^{n}$ are its neighbors. This constraint helps to propagate high-confidence predictions to spatially adjacent regions and reduces noise from isolated outlier predictions. See ‘Model description’ in the Supplementary Note for further details.

### Implementation details

Patch embeddings were extracted using both ResNet-50^57^ (ImageNet^58^ pretrained) and Conch^47^ backbone. Original comparison methods were adapted for multilabel classification where possible. Patch-level predictions were obtained via attention scores (RAMIL, CLAM, DSMIL and so on), Grad-CAM (NIC and NIC-WSS) or instance scores (IAMIL, AddMIL and SMMILe variants without refinement). Only SMMILe’s instance refinement network produces patch-level predictions directly. We used fivefold cross-validation at the patient level, or at the WSI level when patient-level data were unavailable. Random splits used 80% of data for training/validation (90:10) and 20% for testing. All analyses were conducted using Python 3.10.4, with CUDA 11.3.1, OpenSlide 3.4.1 and PyTorch 1.12.1. See ‘Implementation information’ in the Supplementary Note for details.

SMMILe’s compute cost (1.50 GFLOPS, 1.20 M parameters) for 1,024-dimension patch embeddings was comparable to baselines such as ABMIL (0.71 GFLOPS, 0.79 M) and TransMIL (2.70 GFLOPS, 2.67 M); see Supplementary Table 10 and ‘Computational cost’ in the Supplementary Note for details.

### Statistics and reproducibility

No statistical methods were used to predetermine sample sizes, but our sample sizes are comparable to those reported in previous studies^24,25,43,44^ addressing similar tasks. Samples with damaged WSI files, poor image quality or unclear diagnostic labels were excluded from TCGA and in-house datasets before analysis. In all other cases, no data were excluded. All experiments were performed using fivefold cross-validation with random assignment. Patient-level splitting was applied when patient information was available; otherwise, slide-level splitting was used. Data distribution was assumed to be normal, but this was not formally tested. Data collection and analysis were not performed blind to the conditions of the experiments.

### Reporting summary

Further information on research design is available in the Nature Portfolio Reporting Summary linked to this article.

## Supplementary information

Supplementary Information Supplementary Tables 1–10, proofs, synthetic experiment, model description, dataset description, implementation Information, cross-model ablation and computational cost.

Reporting Summary

## Source data

Source Data Fig. 1 Statistical source data.

Source Data Fig. 2 Statistical source data.

Source Data Fig. 4 Statistical source data.

Source Data Fig. 6 Statistical source data.

Source Data Extended Data Fig. 1 Statistical source data.

## Data Availability

The TCGA datasets (TCGA-LU (including LUAD and LUSC), TCGA-RCC and TCGA-STAD), including whole-slide images and diagnostic labels, are publicly available via the Genomic Data Commons portal at https://portal.gdc.cancer.gov. Public datasets, including whole-slide images and spatial annotations used in this study, are available from their respective portals: Camelyon16 (https://camelyon16.grand-challenge.org/), UBC-OCEAN (https://www.kaggle.com/competitions/UBC-OCEAN) and SICAPv2 (https://data.mendeley.com/datasets/9xxm58dvs3/1). Processed spatial annotations, fine-grained subtype labels for TCGA-STAD, and annotations for TCGA-LU and TCGA-RCC are available at https://huggingface.co/datasets/zeyugao/SMMILe_SpatialAnnotation. Extracted patch embeddings, patch-level labels and superpixel segmentation results for all six public datasets are deposited at https://huggingface.co/datasets/zeyugao/SMMILe_Datasets. Two in-house datasets (IH-RCC and IH-ESD) were collected with approval from the Ethics Committee of the First Affiliated Hospital of Xi’an Jiaotong University (KYLLSL2021-420 and KYLLSL2022-333). The processed data (extracted patch embeddings, labels and superpixel segmentation results) are hosted at https://huggingface.co/datasets/zeyugao/SMMILe_PrivateDatasets with gated access enabled. Qualified academic researchers can request access via the repository page. Requests will be reviewed on a case-by-case basis to ensure compliance with ethical and intellectual property obligations. The point of contact for data access is Z.G. (zg323@cam.ac.uk). Approved requests will typically be granted within 4 weeks under a data use agreement. Source data are provided with this paper.

## Code availability

The code for training SMMILe is available via GitHub at https://github.com/ZeyuGaoAi/SMMILe. We have documented the details of model architecture and training. Any additional information is available from the corresponding authors upon request.

## References

[1] Jain, M. S. & Massoud, T. F. Predicting tumour mutational burden from histopathological images using multiscale deep learning. *Nat. Mach. Intell.* **2**, 356–362 (2020).

[2] Lee, Y. et al. Derivation of prognostic contextual histopathological features from whole-slide images of tumours via graph deep learning. *Nat. Biomed. Eng.* **6**, 1452–1466 (2022). 35982331 10.1038/s41551-022-00923-0

[3] Zheng, X. et al. A deep learning model and human-machine fusion for prediction of EBV-associated gastric cancer from histopathology. *Nat. Commun.* **13**, 2790 (2022). 35589792 10.1038/s41467-022-30459-5 PMC9120175

[4] Schmauch, B. et al. A deep learning model to predict RNA-Seq expression of tumours from whole slide images. *Nat. Commun.* **11**, 3877 (2020). 32747659 10.1038/s41467-020-17678-4 PMC7400514

[5] Saldanha, O. L. et al. Self-supervised attention-based deep learning for pan-cancer mutation prediction from histopathology. *npj Precis. Oncol.* **7**, 35 (2023). 36977919 10.1038/s41698-023-00365-0 PMC10050159

[6] Saillard, C. et al. Pacpaint: a histology-based deep learning model uncovers the extensive intratumor molecular heterogeneity of pancreatic adenocarcinoma. *Nat. Commun.* **14**, 3459 (2023). 37311751 10.1038/s41467-023-39026-y PMC10264377

[7] Mandair, D., Reis-Filho, J. S. & Ashworth, A. Biological insights and novel biomarker discovery through deep learning approaches in breast cancer histopathology. *npj Breast Cancer* **9**, 21 (2023). 37024522 10.1038/s41523-023-00518-1 PMC10079681

[8] He, B. et al. Integrating spatial gene expression and breast tumour morphology via deep learning. *Nat. Biomed. Eng.* **4**, 827–834 (2020). 32572199 10.1038/s41551-020-0578-x

[9] Jia, Y., Liu, J., Chen, L., Zhao, T. & Wang, Y. THItoGene: a deep learning method for predicting spatial transcriptomics from histological images. *Brief, Bioinform.* **25**, bbad464 (2024). 10.1093/bib/bbad464 PMC10749789 38145948

[10] Van der Laak, J., Litjens, G. & Ciompi, F. Deep learning in histopathology: the path to the clinic. *Nat. Med.* **27**, 775–784 (2021). 33990804 10.1038/s41591-021-01343-4

[11] Gadermayr, M. & Tschuchnig, M. Multiple instance learning for digital pathology: a review on the state-of-the-art, limitations & future potential. *Comput. Med. Imaging Graph.* **112**, 102337 (2024). 38228020 10.1016/j.compmedimag.2024.102337

[12] Ciga, O., Xu, T. & Martel, A. L. Self supervised contrastive learning for digital histopathology. *Mach. Learn. Appl.* **7**, 100198 (2022).

[13] Wang, X. et al. Transformer-based unsupervised contrastive learning for histopathological image classification. *Med. Image Anal.* **81**, 102559 (2022). 35952419 10.1016/j.media.2022.102559

[14] Chen, R. J. et al. Towards a general-purpose foundation model for computational pathology. *Nat. Med.* **30**, 850–862 (2024). 38504018 10.1038/s41591-024-02857-3 PMC11403354

[15] Xu, H. et al. A whole-slide foundation model for digital pathology from real-world data. *Nature* **630**, 181–188 (2024). 10.1038/s41586-024-07441-w PMC11153137 38778098

[16] Zimmermann, E. et al. Virchow2: scaling self-supervised mixed magnification models in pathology. Preprint at https://arxiv.org/abs/2408.00738 (2024).

[17] Nechaev, D., Pchelnikov, A. & Ivanova, E. Hibou: a family of foundational vision transformers for pathology. Preprint at https://arxiv.org/abs/2406.05074 (2024).

[18] Vorontsov, E. et al. A foundation model for clinical-grade computational pathology and rare cancers detection. *Nat. Med.* **30**, 2924–2935 (2024). 39039250 10.1038/s41591-024-03141-0 PMC11485232

[19] Bouzid, K. et al. Enabling large-scale screening of Barrett’s esophagus using weakly supervised deep learning in histopathology. *Nat. Commun.* **15**, 2026 (2024). 38467600 10.1038/s41467-024-46174-2 PMC10928093

[20] Laleh, N. G. et al. Benchmarking weakly-supervised deep learning pipelines for whole slide classification in computational pathology. *Med. Image Anal.* **79**, 102474 (2022). 35588568 10.1016/j.media.2022.102474

[21] Jiang, R. et al. A transformer-based weakly supervised computational pathology method for clinical-grade diagnosis and molecular marker discovery of gliomas. *Nat. Mach. Intell.* **6**, 876–891 (2024).

[22] El Nahhas, O. S. et al. Regression-based deep-learning predicts molecular biomarkers from pathology slides. *Nat. Commun.* **15**, 1253 (2024). 38341402 10.1038/s41467-024-45589-1 PMC10858881

[23] Ahn, B. et al. Histopathologic image–based deep learning classifier for predicting platinum-based treatment responses in high-grade serous ovarian cancer. *Nat. Commun.* **15**, 4253 (2024). 38762636 10.1038/s41467-024-48667-6 PMC11102549

[24] Li, B., Li, Y. & Eliceiri, K. W. Dual-stream multiple instance learning network for whole slide image classification with self-supervised contrastive learning. *IEEE Conf. Comput. Vis. Pattern Recogn.* **2021**, 14318–14328 (2021). 10.1109/CVPR46437.2021.01409 PMC8765709 35047230

[25] Zhang, H. et al. DTFD-MIL: double-tier feature distillation multiple instance learning for histopathology whole slide image classification. In *IEEE Conference on Computer Vision and Pattern Recognition* 18802–18812 (IEEE, 2022).

[26] Gao, Z. et al. A semi-supervised multi-task learning framework for cancer classification with weak annotation in whole-slide images. *Med. Image Anal.* **83**, 102652 (2023). 36327654 10.1016/j.media.2022.102652

[27] Albuquerque, T., Yüce, A., Herrmann, M. D. & Gomariz, A. Characterizing the interpretability of attention maps in digital pathology. Preprint at https://arxiv.org/abs/2407.02484 (2024).

[28] Javed, S. A. et al. Additive MIL: intrinsically interpretable multiple instance learning for pathology. *Adv. Neural Inf. Process. Syst.* **35**, 20689–20702 (2022).

[29] Kaczmarzyk, J. R., Saltz, J. H. & Koo, P. K. Explainable AI for computational pathology identifies model limitations and tissue biomarkers. Preprint at https://arxiv.org/abs/2409.03080 (2024).

[30] Park, Y. S. et al. A standardized pathology report for gastric cancer. *Journal of Pathology and Translational Medicine* **57**, 1–27 (2023). 36647283 10.4132/jptm.2022.12.23 PMC9846007

[31] Tang, P., Wang, X., Bai, X. & Liu, W. Multiple instance detection network with online instance classifier refinement. In *IEEE Conference on Computer Vision and Pattern Recognition* 2843–2851 (IEEE, 2017).

[32] Ilse, M., Tomczak, J. & Welling, M. Attention-based deep multiple instance learning. In *International Conference on Machine Learning* 2127–2136 (PMLR, 2018).

[33] Bejnordi, B. E. et al. Diagnostic assessment of deep learning algorithms for detection of lymph node metastases in women with breast cancer. *JAMA* **318**, 2199–2210 (2017). 29234806 10.1001/jama.2017.14585 PMC5820737

[34] Cancer Genome Atlas Research Network et al. Comprehensive molecular profiling of lung adenocarcinoma. *Nature* **511**, 543 (2014). 10.1038/nature13385 PMC4231481 25079552

[35] Cancer Genome Atlas Research Network et al. Comprehensive genomic characterization of squamous cell lung cancers. *Nature* **489**, 519 (2012). 10.1038/nature11404 PMC3466113 22960745

[36] Cancer Genome Atlas Research Network et al. Comprehensive molecular characterization of clear cell renal cell carcinoma. *Nature* **499**, 43–49 (2013). 10.1038/nature12222 PMC3771322 23792563

[37] Cancer Genome Atlas Research Network et al. Comprehensive molecular characterization of papillary renal-cell carcinoma. *New Engl. J. Med.* **374**, 135–145 (2016). 10.1056/NEJMoa1505917 PMC4775252 26536169

[38] Davis, C. F. et al. The somatic genomic landscape of chromophobe renal cell carcinoma. *Cancer Cell* **26**, 319–330 (2014). 25155756 10.1016/j.ccr.2014.07.014 PMC4160352

[39] Farahani, H. et al. Deep learning-based histotype diagnosis of ovarian carcinoma whole-slide pathology images. *Mod. Pathol.* **35**, 1983–1990 (2022). 36065012 10.1038/s41379-022-01146-z

[40] Asadi-Aghbolaghi, M. et al. Machine learning-driven histotype diagnosis of ovarian carcinoma: insights from the OCEAN AI challenge. Preprint at *medRxiv*10.1101/2024.04.19.24306099 (2024).

[41] Cancer Genome Atlas Research Network et al. Comprehensive molecular characterization of gastric adenocarcinoma. *Nature* **513**, 202 (2014). 10.1038/nature13480 PMC4170219 25079317

[42] Silva-Rodríguez, J., Colomer, A., Sales, M. A., Molina, R. & Naranjo, V. Going deeper through the Gleason scoring scale: An automatic end-to-end system for histology prostate grading and cribriform pattern detection. *Comput. Methods Programs Biomed.* **195**, 105637 (2020). 32653747 10.1016/j.cmpb.2020.105637

[43] Lu, M. Y. et al. Data-efficient and weakly supervised computational pathology on whole-slide images. *Nat. Biomed. Eng.* **5**, 555–570 (2021). 33649564 10.1038/s41551-020-00682-w PMC8711640

[44] Shao, Z. et al. Transmil: Transformer based correlated multiple instance learning for whole slide image classification. *Adv. Neural Inf. Process. Syst.* **34**, 2136–2147 (2021).

[45] Tellez, D., Litjens, G., Van Der Laak, J. & Ciompi, F. Neural image compression for gigapixel histopathology image analysis. *IEEE Trans. Pattern Anal. Mach. Intell.* **43**, 567–578 (2021). 31442971 10.1109/TPAMI.2019.2936841

[46] Chikontwe, P. et al. Weakly supervised segmentation on neural compressed histopathology with self-equivariant regularization. *Med. Image Anal.* **80**, 102482 (2022). 35688048 10.1016/j.media.2022.102482

[47] Lu, M. Y. et al. A visual-language foundation model for computational pathology. *Nat. Med.* **30**, 863–874 (2024). 38504017 10.1038/s41591-024-02856-4 PMC11384335

[48] Selvaraju, R. R. et al. Grad-cam: visual explanations from deep networks via gradient-based localization. In *Proc. IEEE International Conference on Computer Vision* 618–626 (IEEE, 2017).

[49] Kraus, O. Z., Ba, J. L. & Frey, B. J. Classifying and segmenting microscopy images with deep multiple instance learning. *Bioinformatics* **32**, i52–i59 (2016). 27307644 10.1093/bioinformatics/btw252 PMC4908336

[50] Das, K., Conjeti, S., Roy, A. G., Chatterjee, J. & Sheet, D. Multiple instance learning of deep convolutional neural networks for breast histopathology whole slide classification. In *IEEE International Symposium on Biomedical Imaging* 578–581 (IEEE, 2018).

[51] Campanella, G. et al. Clinical-grade computational pathology using weakly supervised deep learning on whole slide images. *Nat. Med.* **25**, 1301–1309 (2019). 31308507 10.1038/s41591-019-0508-1 PMC7418463

[52] Yao, J., Zhu, X. & Huang, J. Deep multi-instance learning for survival prediction from whole slide images. In *International Conference on Medical Image Computing and Computer Assisted Intervention* 496–504 (Springer, 2019).

[53] Salahuddin, Z., Woodruff, H. C., Chatterjee, A. & Lambin, P. Transparency of deep neural networks for medical image analysis: a review of interpretability methods. *Comput. Biol. Med.* **140**, 105111 (2022). 34891095 10.1016/j.compbiomed.2021.105111

[54] Wang, X., Yan, Y., Tang, P., Bai, X. & Liu, W. Revisiting multiple instance neural networks. *Pattern Recogn.* **74**, 15–24 (2018).

[55] Sun, M., Han, T. X., Liu, M.-C. & Khodayari-Rostamabad, A. Multiple instance learning convolutional neural networks for object recognition. In *International Conference on Pattern Recognition* 3270–3275 (IEEE, 2016).

[56] Achanta, R. et al. SLIC superpixels compared to state-of-the-art superpixel methods. *IEEE Trans. Pattern Anal. Mach. Intell.* **34**, 2274–2282 (2012). 22641706 10.1109/TPAMI.2012.120

[57] He, K., Zhang, X., Ren, S. & Sun, J. Deep residual learning for image recognition. In *IEEE Conference on Computer Vision and Pattern Recognition* 770–778 (IEEE, 2016).

[58] Deng, J. et al. ImageNet: a large-scale hierarchical image database. In *IEEE Conference on Computer Vision and Pattern Recognition* 248–255 (IEEE, 2009).

