# Supplementary material for: SMMILe enables accurate spatial quantification in digital pathology using multiple-instance learning
Source: Nat Cancer. 2025 Nov 19;6(12):2025–41. doi: 10.1038/s43018-025-01060-8 (PMC12727519; doi:10.1038/s43018-025-01060-8)
Supplement: Supplementary file 1 — Supplementary Tables 1–10, proofs, synthetic experiment, model description, dataset description, implementation Information, cross-model ablation and computational cost. [file 43018_2025_1060_MOESM1_ESM.pdf]

nature cancer

Supplementary information https://doi.org/10.1038/s43018-025-01060-8

SMMILe enables accurate spatial quantification in digital pathology using multiple-instance learning

In the format provided by the authors and unedited

The multiple instance learning-based tool SMMILe achieves accurate spatial quantification in digital pathology: Supplementary notes

Setting	Value
Number of Classes	2
Embedding Dimension of Each Instance	3
Positive Distribution (Class 0)	Norm(-3.0,0.5);Norm(-2.0,0.5);Norm(3.0,0.5)
Positive Distribution (Class 1)	Norm(-2.0,0.5);Norm(-3.0,0.5);Norm(3.0,0.5)
Negative Distribution	Norm(1.0,0.5);Norm(-2.5,0.5);Norm(2.0,0.5)
Number of Bags per Class	200
Number of Instances per Bag	1000
The Ratio of Positive Instance per Bag	[0.1,1.0]
Attention Mechanism	Gated
Attention Network Feature Dimensions	8, 4
Dropout Rate	0.25
Optimizer	Adam
Learning Rate	1e-3
Weight Decay	1e-5
Loss Function	Binary cross-entropy
Stop Condition	10 epochs without loss decrease

Supplementary Table 1: Settings for Synthetic Dataset Generation and Experimental Model Training

Encoder	Dataset	RAMIL	IAMIL	CLAM	DSMIL	TransMIL	DTFD	AddMIL	NIC	NICWSS	SMMILe
ResNet-50	Breast	93.24±3.60	94.82±2.84	93.76±2.95	75.41±3.38	91.42±1.85	93.37±3.22	91.62±4.26	56.17±8.75	53.88±8.49	**95.24±2.05**
	Lung	93.55±3.22	94.59±2.55	94.51±2.28	90.60±2.83	91.54±2.29	92.38±3.63	93.40±2.90	85.94±4.17	86.09±4.43	**94.91±1.89**
	Renal-3	98.75±0.71	99.00±0.51	99.01±0.74	97.95±1.17	98.61±0.75	98.65±0.85	98.74±0.72	98.44±0.62	96.69±2.20	**99.24±0.49**
	Renal-4	97.21±1.08	93.35±1.99	97.29±1.43	95.34±1.47	97.35±0.84	96.25±1.76	96.90±1.78	96.06±1.78	96.41±2.18	**98.29±0.58**
	Ovarian	92.60±0.65	91.92±1.87	91.82±2.50	88.17±2.17	91.18±1.99	89.26±1.44	91.16±2.58	78.77±6.06	84.09±5.83	**94.11 ±1.13**
	Gastric	87.21 ±3.09	82.76 ±2.30	88.06 ±3.15	85.92 ±4.20	85.59 ±4.39	81.01 ±2.89	83.14 ±2.04	80.19 ±4.59	79.18 ±6.98	**88.12±2.73**
	Prostate	88.02±1.96	86.80±1.50	86.71±1.46	87.74±2.59	85.61±6.79	83.44±2.96	86.62±2.19	66.82±3.30	61.14±10.84	**90.92±2.68**
	Gastric Endo.	71.43±10.41	81.57±4.14	72.81±7.09	73.12±18.99	77.30±8.94	70.27±6.25	77.87±9.82	78.71±7.53	76.42±6.66	**92.75±3.59**
Conch	Breast	99.45±0.67	98.84±1.32	99.14±1.13	99.16±1.07	**99.55±0.66**	99.42±0.69	99.21±1.17	90.71±5.46	91.43±6.05	99.35±0.86
	Lung	97.50±1.40	97.52±0.98	97.48±1.25	97.38±1.44	**97.73±0.84**	97.14±1.39	97.60±1.06	96.27±1.69	96.69±1.41	97.26±1.06
	Renal-3	99.70±0.16	99.67±0.15	99.64±0.20	99.64±0.25	99.59±0.25	99.67±0.17	99.70±0.12	99.43±0.28	99.49±0.35	**99.71±0.19**
	Renal-4	99.23±0.61	97.72±2.25	99.30±0.54	99.01±0.80	98.85±0.92	98.58±0.75	99.04±1.00	98.48±1.02	97.71±0.70	**98.73±0.96**
	Ovarian	97.22±1.59	96.80±0.68	97.03±1.22	97.16±1.10	**97.17±0.91**	96.96±1.50	97.27±1.16	93.82±1.49	95.33±0.40	97.01±0.44
	Gastric	92.92±2.24	**93.23±2.19**	92.86±2.16	91.47±0.92	88.27±0.89	89.01±1.97	91.19±2.01	82.87±2.16	84.51±1.38	92.70±0.69
	Gleason	93.68±1.34	94.64±0.55	93.50±1.39	93.75±1.52	91.67±2.87	90.42±2.84	93.13±1.26	76.88±3.16	84.03±1.86	**94.64±0.79**
	Gastric Endo.	97.54±3.53	96.86±3.27	97.22±3.64	97.31±4.31	92.45±3.90	87.51±4.12	97.10±3.28	81.47±3.52	79.31±9.86	**98.01±2.25**

Supplementary Table 2: The comparison results of WSI classification (macro AUC score, present in %). The best results are highlighted in boldface

Encoder	Dataset	RAMIL	IAMIL	CLAM	DSMIL	TransMIL	DTFD	AddMIL	NIC	NICWSS	SMMILe
ResNet-50	Breast	$75.12_{\pm 8.18}$	$79.45_{\pm 8.27}$	$74.84_{\pm 8.40}$	$90.26_{\pm 5.85}$	$52.02_{\pm 7.37}$	$81.81_{\pm 4.51}$	$68.66_{\pm 12.16}$	$45.93_{\pm 5.14}$	$51.78_{\pm 11.72}$	$\mathbf{91.25_{\pm 3.73}}$
	Lung	$81.19_{\pm 2.66}$	$78.32_{\pm 0.94}$	$82.73_{\pm 2.63}$	$62.10_{\pm 3.35}$	$47.90_{\pm 3.47}$	$81.46_{\pm 2.85}$	$54.94_{\pm 2.51}$	$58.45_{\pm 2.41}$	$57.71_{\pm 2.67}$	$\mathbf{87.33_{\pm 1.49}}$
	Renal-3	$79.49_{\pm 1.36}$	$78.64_{\pm 2.72}$	$79.92_{\pm 0.83}$	$77.26_{\pm 2.32}$	$51.44_{\pm 2.48}$	$81.44_{\pm 0.45}$	$59.82_{\pm 5.71}$	$75.74_{\pm 1.68}$	$72.43_{\pm 5.75}$	$\mathbf{88.28_{\pm 1.08}}$
	Renal-4	$85.01_{\pm 1.95}$	$56.39_{\pm 2.62}$	$82.43_{\pm 2.96}$	$75.12_{\pm 1.49}$	$51.65_{\pm 4.56}$	$85.68_{\pm 1.20}$	$56.23_{\pm 1.77}$	$79.81_{\pm 3.29}$	$81.83_{\pm 1.59}$	$\mathbf{89.73_{\pm 1.12}}$
	Ovarian	$85.83_{\pm 3.42}$	$82.97_{\pm 5.95}$	$85.28_{\pm 5.07}$	$63.62_{\pm 6.01}$	$45.17_{\pm 2.19}$	$89.81_{\pm 4.51}$	$54.58_{\pm 5.11}$	$63.91_{\pm 8.10}$	$69.13_{\pm 3.73}$	$\mathbf{94.40_{\pm 4.02}}$
	Gastric	$75.69_{\pm 3.20}$	$54.71_{\pm 7.49}$	$74.82_{\pm 4.04}$	$81.84_{\pm 2.95}$	NaN	$48.02_{\pm 8.76}$	$40.37_{\pm 6.14}$	$80.22_{\pm 1.85}$	$73.02_{\pm 4.22}$	$\mathbf{88.92_{\pm 3.22}}$
	Prostate	$63.68_{\pm 2.90}$	$65.65_{\pm 2.50}$	$62.49_{\pm 4.33}$	$70.88_{\pm 1.78}$	NaN	$56.80_{\pm 4.32}$	$50.85_{\pm 4.76}$	$52.71_{\pm 3.85}$	$57.28_{\pm 8.00}$	$\mathbf{81.70_{\pm 2.15}}$
	Gastric Endo.	$61.16_{\pm 7.97}$	$68.46_{\pm 10.78}$	$57.40_{\pm 14.19}$	$53.95_{\pm 1.90}$	NaN	$47.09_{\pm 4.72}$	$50.04_{\pm 4.41}$	$58.09_{\pm 4.95}$	$57.76_{\pm 3.66}$	$\mathbf{79.07_{\pm 3.92}}$
Conch	Breast	$97.47_{\pm 1.24}$	$96.28_{\pm 2.44}$	$96.77_{\pm 1.39}$	$97.92_{\pm 0.83}$	$89.06_{\pm 3.52}$	$96.79_{\pm 1.19}$	$75.11_{\pm 11.16}$	$96.61_{\pm 0.80}$	$97.40_{\pm 2.14}$	$\mathbf{98.31_{\pm 0.85}}$
	Lung	$89.26_{\pm 2.43}$	$83.77_{\pm 2.55}$	$87.96_{\pm 1.89}$	$79.67_{\pm 1.51}$	$66.44_{\pm 6.22}$	$89.05_{\pm 2.85}$	$67.90_{\pm 4.27}$	$82.10_{\pm 1.83}$	$83.14_{\pm 2.16}$	$\mathbf{92.62_{\pm 1.33}}$
	Renal-3	$89.22_{\pm 2.39}$	$86.77_{\pm 4.26}$	$90.51_{\pm 2.19}$	$86.71_{\pm 2.39}$	$83.53_{\pm 8.20}$	$87.64_{\pm 3.00}$	$73.57_{\pm 3.95}$	$90.55_{\pm 2.35}$	$90.59_{\pm 2.18}$	$\mathbf{93.62_{\pm 1.24}}$
	Renal-4	$90.38_{\pm 0.66}$	$89.32_{\pm 1.87}$	$90.14_{\pm 1.09}$	$82.95_{\pm 2.19}$	$73.05_{\pm 14.31}$	$91.16_{\pm 0.88}$	$77.19_{\pm 2.95}$	$86.55_{\pm 0.52}$	$86.45_{\pm 1.91}$	$\mathbf{94.36_{\pm 1.00}}$
	Ovarian	$93.65_{\pm 1.97}$	$94.18_{\pm 2.03}$	$94.23_{\pm 1.90}$	$81.43_{\pm 3.62}$	$74.75_{\pm 12.64}$	$95.53_{\pm 0.89}$	$71.14_{\pm 6.30}$	$75.76_{\pm 7.35}$	$77.15_{\pm 8.78}$	$\mathbf{96.67_{\pm 1.39}}$
	Gastric	$89.56_{\pm 1.86}$	$90.31_{\pm 1.14}$	$89.96_{\pm 1.54}$	$89.09_{\pm 2.05}$	NaN	$79.32_{\pm 4.80}$	$49.43_{\pm 6.71}$	$83.27_{\pm 4.55}$	$85.75_{\pm 3.20}$	$\mathbf{94.77_{\pm 0.87}}$
	Prostate	$77.51_{\pm 3.01}$	$80.16_{\pm 2.95}$	$77.08_{\pm 2.30}$	$77.97_{\pm 3.46}$	NaN	$74.52_{\pm 8.65}$	$60.18_{\pm 6.63}$	$71.41_{\pm 2.85}$	$78.72_{\pm 1.33}$	$\mathbf{84.09_{\pm 2.48}}$
	Gastric Endo.	$79.71_{\pm 4.25}$	$81.80_{\pm 1.68}$	$79.77_{\pm 3.79}$	$62.80_{\pm 3.22}$	NaN	$59.15_{\pm 5.60}$	$50.26_{\pm 2.00}$	$63.55_{\pm 3.49}$	$67.60_{\pm 7.10}$	$\mathbf{84.57_{\pm 3.83}}$

Supplementary Table 3: The comparison results of spatial quantification (macro AUC score, presented in %). The best results are highlighted in bold.

Encoder	Dataset	RAMIL	IAMIL	CLAM	DSMIL	TransMIL	DTFD	AddMIL	NIC	NICWSS	SMMILe
ResNet-50	Breast	$18.61_{\pm 8.84}$	$84.25_{\pm 18.35}$	$19.45_{\pm 12.93}$	$85.32_{\pm 22.98}$	$2.08_{\pm 2.66}$	$24.78_{\pm 12.36}$	$2.04_{\pm 3.39}$	$2.56_{\pm 2.10}$	$12.40_{\pm 23.12}$	$\mathbf{90.72_{\pm 4.80}}$
	Lung	$84.52_{\pm 2.38}$	$\mathbf{92.15_{\pm 2.23}}$	$84.21_{\pm 1.54}$	$59.23_{\pm 3.31}$	$46.78_{\pm 5.98}$	$83.57_{\pm 2.65}$	$60.63_{\pm 8.92}$	$65.94_{\pm 2.19}$	$64.58_{\pm 2.09}$	$81.26_{\pm 2.63}$
	Renal-3	$76.81_{\pm 3.46}$	$\mathbf{92.38_{\pm 4.82}}$	$80.00_{\pm 1.72}$	$63.64_{\pm 4.84}$	$49.26_{\pm 10.59}$	$81.32_{\pm 1.78}$	$62.67_{\pm 27.74}$	$74.63_{\pm 2.90}$	$73.21_{\pm 3.39}$	$82.32_{\pm 2.03}$
	Renal-4	$81.83_{\pm 3.40}$	$58.29_{\pm 7.97}$	$74.86_{\pm 7.77}$	$65.81_{\pm 3.67}$	$43.63_{\pm 11.39}$	$80.20_{\pm 4.62}$	$64.48_{\pm 14.80}$	$79.42_{\pm 4.49}$	$79.86_{\pm 2.15}$	$\mathbf{88.06_{\pm 2.30}}$
	Ovarian	$95.24_{\pm 0.79}$	$\mathbf{100.00_{\pm 0.00}}$	$96.82_{\pm 0.50}$	$94.11_{\pm 1.12}$	$85.22_{\pm 3.63}$	$97.78_{\pm 0.85}$	$97.20_{\pm 5.47}$	$96.36_{\pm 1.43}$	$96.15_{\pm 1.16}$	$97.39_{\pm 0.37}$
	Gastric	$65.01_{\pm 8.28}$	$47.41_{\pm 12.48}$	$63.29_{\pm 7.64}$	$61.66_{\pm 5.78}$	NaN	$63.98_{\pm 5.76}$	$25.85_{\pm 9.85}$	$68.75_{\pm 8.06}$	$58.57_{\pm 9.42}$	$\mathbf{80.27_{\pm 4.50}}$
	Prostate	$54.33_{\pm 11.38}$	$58.80_{\pm 2.70}$	$54.89_{\pm 6.15}$	$63.15_{\pm 5.94}$	NaN	$\mathbf{74.56_{\pm 2.57}}$	$44.47_{\pm 11.27}$	$57.52_{\pm 10.27}$	$45.07_{\pm 9.21}$	$70.08_{\pm 3.23}$
	Gastric-Endo.	$45.94_{\pm 4.82}$	$61.14_{\pm 10.18}$	$41.78_{\pm 10.64}$	$42.03_{\pm 1.20}$	NaN	$60.04_{\pm 2.97}$	$36.98_{\pm 2.96}$	$42.40_{\pm 4.61}$	$41.76_{\pm 3.52}$	$\mathbf{61.40_{\pm 2.92}}$
Conch	Breast	$92.77_{\pm 5.76}$	$86.05_{\pm 21.37}$	$92.75_{\pm 5.60}$	$\mathbf{94.76_{\pm 4.26}}$	$53.97_{\pm 20.35}$	$92.68_{\pm 5.34}$	$48.06_{\pm 45.75}$	$78.62_{\pm 4.75}$	$86.27_{\pm 5.64}$	$93.53_{\pm 5.29}$
	Lung	$91.21_{\pm 2.66}$	$\mathbf{94.98_{\pm 1.60}}$	$89.57_{\pm 3.33}$	$67.46_{\pm 2.29}$	$56.57_{\pm 4.99}$	$91.81_{\pm 1.76}$	$84.37_{\pm 5.81}$	$90.98_{\pm 3.40}$	$91.75_{\pm 3.30}$	$91.48_{\pm 1.16}$
	Renal-3	$88.33_{\pm 3.34}$	$\mathbf{92.53_{\pm 3.55}}$	$89.01_{\pm 3.02}$	$68.98_{\pm 3.20}$	$87.42_{\pm 9.53}$	$87.23_{\pm 4.86}$	$67.39_{\pm 19.38}$	$91.53_{\pm 2.09}$	$91.50_{\pm 2.55}$	$90.64_{\pm 2.45}$
	Renal-4	$90.38_{\pm 1.05}$	$\mathbf{96.99_{\pm 1.61}}$	$89.56_{\pm 2.44}$	$73.86_{\pm 3.72}$	$81.29_{\pm 11.93}$	$91.36_{\pm 2.51}$	$88.35_{\pm 18.14}$	$92.03_{\pm 1.32}$	$92.37_{\pm 2.06}$	$88.20_{\pm 2.34}$
	Ovarian	$98.75_{\pm 0.27}$	$\mathbf{100.00_{\pm 0.00}}$	$98.69_{\pm 0.32}$	$96.52_{\pm 1.11}$	$96.98_{\pm 3.13}$	$99.12_{\pm 0.14}$	$96.89_{\pm 4.36}$	$98.19_{\pm 0.48}$	$97.64_{\pm 0.64}$	$98.70_{\pm 0.44}$
	Gastric	$78.44_{\pm 6.29}$	$78.81_{\pm 1.46}$	$79.05_{\pm 6.56}$	$73.20_{\pm 5.37}$	NaN	$69.61_{\pm 9.93}$	$39.59_{\pm 13.10}$	$73.76_{\pm 3.40}$	$76.07_{\pm 2.66}$	$\mathbf{86.36_{\pm 2.98}}$
	Prostate	$63.34_{\pm 3.45}$	$68.45_{\pm 5.04}$	$62.11_{\pm 3.30}$	$62.98_{\pm 4.58}$	NaN	$61.19_{\pm 7.46}$	$49.89_{\pm 9.94}$	$60.05_{\pm 3.52}$	$67.09_{\pm 2.71}$	$\mathbf{74.17_{\pm 3.15}}$
	Gastric-Endo.	$68.97_{\pm 5.94}$	$\mathbf{70.01_{\pm 3.16}}$	$69.68_{\pm 4.68}$	$48.70_{\pm 0.65}$	NaN	$62.98_{\pm 5.76}$	$35.36_{\pm 3.35}$	$47.16_{\pm 3.06}$	$52.45_{\pm 8.16}$	$69.85_{\pm 2.21}$

Supplementary Table 4: The comparison results of spatial quantification (macro precision, presented in %). The best results are highlighted in bold.

Encoder	Dataset	RAMIL	IAMIL	CLAM	DSMIL	TransMIL	DTFD	AddMIL	NIC	NICWSS	SMMILe
ResNet-50	Breast	$53.15_{\pm 13.29}$	$1.21_{\pm 0.70}$	$53.89_{\pm 12.84}$	$29.93_{\pm 16.92}$	$2.89_{\pm 6.40}$	$\mathbf{55.88_{\pm 4.75}}$	$4.47_{\pm 6.85}$	$0.73_{\pm 0.72}$	$5.02_{\pm 10.14}$	$53.07_{\pm 12.67}$
	Lung	$57.61_{\pm 3.48}$	$0.56_{\pm 0.12}$	$63.51_{\pm 6.09}$	$77.06_{\pm 1.50}$	$8.51_{\pm 6.30}$	$59.18_{\pm 8.24}$	$11.43_{\pm 9.98}$	$25.41_{\pm 2.99}$	$26.76_{\pm 4.32}$	$\mathbf{82.31_{\pm 3.09}}$
	Renal-3	$65.59_{\pm 2.76}$	$1.05_{\pm 0.38}$	$59.70_{\pm 2.76}$	$81.83_{\pm 1.10}$	$12.88_{\pm 11.72}$	$63.45_{\pm 2.29}$	$1.61_{\pm 1.35}$	$55.44_{\pm 2.40}$	$48.67_{\pm 9.47}$	$\mathbf{80.17_{\pm 2.03}}$
	Renal-4	$68.56_{\pm 2.60}$	$0.23_{\pm 0.03}$	$63.54_{\pm 4.69}$	$54.51_{\pm 2.39}$	$19.32_{\pm 17.72}$	$68.84_{\pm 4.26}$	$2.50_{\pm 1.34}$	$53.43_{\pm 5.03}$	$59.37_{\pm 1.98}$	$\mathbf{79.73_{\pm 2.47}}$
	Ovarian	$72.16_{\pm 3.26}$	$0.38_{\pm 0.12}$	$74.88_{\pm 9.09}$	$43.65_{\pm 9.97}$	$21.83_{\pm 8.22}$	$77.96_{\pm 12.25}$	$5.34_{\pm 7.56}$	$21.96_{\pm 16.56}$	$42.92_{\pm 12.45}$	$\mathbf{93.34_{\pm 4.60}}$
	Gastric	$61.36_{\pm 4.67}$	$25.49_{\pm 0.38}$	$63.49_{\pm 1.74}$	$62.02_{\pm 6.29}$	NaN	$29.81_{\pm 4.61}$	$27.76_{\pm 9.90}$	$51.90_{\pm 2.76}$	$45.16_{\pm 9.39}$	$\mathbf{74.28_{\pm 4.82}}$
	Prostate	$35.83_{\pm 2.44}$	$27.24_{\pm 0.50}$	$36.44_{\pm 1.98}$	$52.39_{\pm 3.07}$	NaN	$33.06_{\pm 1.58}$	$31.12_{\pm 3.78}$	$28.92_{\pm 3.16}$	$43.58_{\pm 11.17}$	$\mathbf{69.43_{\pm 4.59}}$
	Gastric-Endo.	$43.04_{\pm 4.20}$	$34.03_{\pm 0.32}$	$42.10_{\pm 10.58}$	$40.10_{\pm 1.44}$	NaN	$31.62_{\pm 2.29}$	$42.25_{\pm 6.44}$	$40.37_{\pm 4.29}$	$38.97_{\pm 1.99}$	$\mathbf{64.86_{\pm 4.00}}$
Conch	Breast	$83.55_{\pm 7.02}$	$3.81_{\pm 5.08}$	$81.50_{\pm 9.37}$	$73.66_{\pm 7.70}$	$5.83_{\pm 9.33}$	$82.82_{\pm 2.80}$	$1.39_{\pm 0.68}$	$65.16_{\pm 3.98}$	$75.73_{\pm 4.27}$	$\mathbf{84.62_{\pm 6.01}}$
	Lung	$74.23_{\pm 6.07}$	$3.18_{\pm 1.71}$	$74.98_{\pm 7.76}$	$87.19_{\pm 1.50}$	$30.15_{\pm 14.90}$	$70.40_{\pm 8.17}$	$6.49_{\pm 3.49}$	$47.52_{\pm 3.10}$	$51.46_{\pm 2.92}$	$\mathbf{85.18_{\pm 1.30}}$
	Renal-3	$81.27_{\pm 2.61}$	$1.95_{\pm 1.63}$	$81.44_{\pm 7.06}$	$\mathbf{92.00_{\pm 1.63}}$	$54.78_{\pm 9.44}$	$77.02_{\pm 5.78}$	$5.12_{\pm 2.21}$	$70.24_{\pm 4.04}$	$71.52_{\pm 3.63}$	$86.28_{\pm 3.74}$
	Renal-4	$77.56_{\pm 3.37}$	$7.01_{\pm 5.96}$	$78.22_{\pm 5.67}$	$85.44_{\pm 1.79}$	$37.86_{\pm 21.39}$	$74.11_{\pm 10.14}$	$2.65_{\pm 2.10}$	$63.55_{\pm 1.34}$	$66.24_{\pm 2.54}$	$\mathbf{91.56_{\pm 2.78}}$
	Ovarian	$88.26_{\pm 3.75}$	$0.88_{\pm 0.73}$	$88.84_{\pm 1.91}$	$77.80_{\pm 3.42}$	$17.63_{\pm 11.05}$	$84.03_{\pm 4.05}$	$2.26_{\pm 3.17}$	$49.33_{\pm 10.93}$	$56.99_{\pm 6.00}$	$\mathbf{93.51_{\pm 2.39}}$
	Gastric	$77.73_{\pm 3.36}$	$30.09_{\pm 3.57}$	$78.01_{\pm 2.99}$	$69.85_{\pm 5.68}$	NaN	$66.31_{\pm 2.69}$	$27.69_{\pm 2.33}$	$62.51_{\pm 6.53}$	$65.81_{\pm 5.57}$	$\mathbf{83.90_{\pm 4.74}}$
	Prostate	$58.70_{\pm 3.31}$	$28.51_{\pm 0.60}$	$57.23_{\pm 2.95}$	$66.74_{\pm 5.92}$	NaN	$56.32_{\pm 8.95}$	$36.63_{\pm 6.51}$	$44.50_{\pm 2.93}$	$52.21_{\pm 4.42}$	$\mathbf{73.11_{\pm 2.05}}$
	Gastric-Endo.	$59.82_{\pm 4.09}$	$34.71_{\pm 0.40}$	$59.87_{\pm 3.13}$	$44.51_{\pm 2.96}$	NaN	$34.13_{\pm 3.32}$	$34.46_{\pm 3.32}$	$44.84_{\pm 3.90}$	$48.55_{\pm 7.19}$	$\mathbf{72.10_{\pm 4.69}}$

Supplementary Table 5: The comparison results of spatial quantification (macro recall, presented in %). The best results are highlighted in bold.

Encoder	Dataset	RAMIL	IAMIL	CLAM	DSMIL	TransMIL	DTFD	AddMIL	NIC	NICWSS	SMMILe
ResNet-50	Breast	26.77$_{\pm10.72}$	2.37$_{\pm1.37}$	27.04$_{\pm13.33}$	42.91$_{\pm19.53}$	1.81$_{\pm3.93}$	32.74$_{\pm12.84}$	2.78$_{\pm4.54}$	1.00$_{\pm0.81}$	7.09$_{\pm14.13}$	**66.47$_{\pm10.21}$**
	Lung	68.49$_{\pm2.98}$	1.10$_{\pm0.24}$	72.31$_{\pm4.45}$	66.93$_{\pm2.06}$	13.69$_{\pm9.04}$	68.99$_{\pm5.62}$	17.74$_{\pm12.12}$	36.61$_{\pm3.29}$	37.71$_{\pm4.44}$	**81.72$_{\pm1.51}$**
	Renal-3	70.69$_{\pm1.99}$	2.07$_{\pm0.75}$	68.36$_{\pm2.21}$	71.51$_{\pm3.00}$	17.92$_{\pm14.66}$	71.26$_{\pm1.65}$	3.07$_{\pm2.49}$	63.60$_{\pm2.32}$	58.20$_{\pm7.99}$	**81.20$_{\pm1.06}$**
	Renal-4	74.57$_{\pm2.21}$	0.46$_{\pm0.06}$	68.44$_{\pm3.57}$	59.60$_{\pm2.62}$	23.51$_{\pm14.63}$	73.90$_{\pm1.70}$	4.77$_{\pm2.51}$	63.75$_{\pm3.88}$	68.08$_{\pm1.49}$	**83.64$_{\pm1.10}$**
	Ovarian	82.07$_{\pm2.06}$	0.75$_{\pm0.23}$	84.23$_{\pm5.71}$	59.09$_{\pm10.16}$	34.18$_{\pm10.81}$	86.29$_{\pm7.68}$	9.27$_{\pm12.83}$	33.22$_{\pm22.63}$	58.37$_{\pm12.70}$	**95.27$_{\pm2.52}$**
	Gastric	60.98$_{\pm6.68}$	14.31$_{\pm1.49}$	60.37$_{\pm6.33}$	57.56$_{\pm6.58}$	NaN	17.73$_{\pm5.55}$	19.57$_{\pm5.93}$	53.98$_{\pm3.17}$	45.50$_{\pm12.55}$	**76.55$_{\pm4.55}$**
	Prostate	35.14$_{\pm2.04}$	21.62$_{\pm2.52}$	36.79$_{\pm2.21}$	52.34$_{\pm4.00}$	NaN	26.62$_{\pm1.96}$	26.97$_{\pm5.06}$	24.54$_{\pm5.17}$	43.22$_{\pm10.16}$	**67.62$_{\pm4.66}$**
	Gastric-Endo.	40.37$_{\pm3.41}$	19.26$_{\pm1.88}$	39.27$_{\pm9.61}$	40.19$_{\pm1.88}$	NaN	24.78$_{\pm3.77}$	31.74$_{\pm3.15}$	39.48$_{\pm3.94}$	38.60$_{\pm2.70}$	**61.75$_{\pm2.60}$**
Conch	Breast	87.67$_{\pm4.13}$	6.98$_{\pm8.91}$	86.34$_{\pm4.88}$	82.69$_{\pm5.25}$	7.54$_{\pm10.54}$	87.36$_{\pm1.96}$	2.55$_{\pm1.52}$	71.16$_{\pm3.12}$	80.64$_{\pm4.73}$	**88.71$_{\pm4.12}$**
	Lung	81.73$_{\pm3.91}$	6.12$_{\pm3.17}$	81.33$_{\pm3.71}$	76.03$_{\pm1.10}$	37.77$_{\pm14.07}$	79.49$_{\pm5.53}$	11.84$_{\pm5.85}$	62.33$_{\pm2.08}$	65.86$_{\pm1.99}$	**88.21$_{\pm0.93}$**
	Renal-3	84.64$_{\pm2.79}$	3.78$_{\pm3.08}$	84.97$_{\pm4.80}$	78.83$_{\pm2.54}$	66.86$_{\pm7.71}$	81.70$_{\pm4.39}$	9.47$_{\pm3.95}$	79.45$_{\pm3.06}$	80.26$_{\pm3.02}$	**89.77$_{\pm1.85}$**
	Renal-4	83.44$_{\pm1.84}$	12.63$_{\pm10.07}$	83.38$_{\pm2.98}$	79.19$_{\pm2.51}$	49.58$_{\pm23.55}$	81.43$_{\pm5.91}$	5.09$_{\pm3.91}$	75.18$_{\pm1.29}$	77.10$_{\pm1.28}$	**89.81$_{\pm1.46}$**
	Ovarian	93.17$_{\pm2.08}$	1.73$_{\pm1.43}$	93.50$_{\pm1.15}$	86.12$_{\pm2.11}$	28.55$_{\pm16.19}$	90.91$_{\pm2.37}$	4.26$_{\pm5.80}$	65.11$_{\pm9.59}$	71.83$_{\pm4.73}$	**96.02$_{\pm1.28}$**
	Gastric	77.14$_{\pm4.52}$	21.63$_{\pm4.39}$	77.61$_{\pm4.48}$	68.88$_{\pm4.64}$	NaN	45.40$_{\pm8.62}$	19.86$_{\pm2.08}$	65.73$_{\pm5.62}$	69.15$_{\pm4.73}$	**84.62$_{\pm2.63}$**
	Prostate	59.78$_{\pm2.93}$	23.84$_{\pm2.18}$	58.21$_{\pm2.53}$	63.69$_{\pm5.62}$	NaN	52.98$_{\pm6.52}$	32.43$_{\pm3.21}$	46.75$_{\pm3.37}$	55.44$_{\pm4.40}$	**71.84$_{\pm2.32}$**
	Gastric-Endo.	61.10$_{\pm4.94}$	20.39$_{\pm1.90}$	61.20$_{\pm3.57}$	42.20$_{\pm1.62}$	NaN	38.78$_{\pm4.99}$	31.92$_{\pm1.48}$	45.17$_{\pm3.76}$	48.08$_{\pm7.69}$	**69.77$_{\pm2.39}$**

Supplementary Table 6: The comparison results of spatial quantification (macro F1 score, presented in %). The best results are highlighted in bold.

Encoder	Dataset	RAMIL	IAMIL	CLAM	DSMIL	TransMIL	DTFD	AddMIL	NIC	NICWSS	SMMILe
ResNet-50	Breast	79.79$_{\pm4.79}$	93.09$_{\pm3.00}$	79.11$_{\pm6.82}$	94.83$_{\pm2.75}$	91.40$_{\pm6.08}$	83.26$_{\pm4.24}$	78.86$_{\pm11.95}$	91.14$_{\pm3.09}$	91.88$_{\pm2.72}$	**95.52$_{\pm2.48}$**
	Lung	71.37$_{\pm2.04}$	46.08$_{\pm2.82}$	73.87$_{\pm3.03}$	58.78$_{\pm2.40}$	45.45$_{\pm2.66}$	71.62$_{\pm2.84}$	47.76$_{\pm3.87}$	52.50$_{\pm2.02}$	52.41$_{\pm2.42}$	**80.13$_{\pm1.22}$**
	Renal-3	73.08$_{\pm1.27}$	50.83$_{\pm2.53}$	72.64$_{\pm1.24}$	67.67$_{\pm3.25}$	49.58$_{\pm1.85}$	74.66$_{\pm0.56}$	50.32$_{\pm2.37}$	68.56$_{\pm1.48}$	65.86$_{\pm4.65}$	**81.09$_{\pm1.06}$**
	Renal-4	80.41$_{\pm1.99}$	43.53$_{\pm2.19}$	75.54$_{\pm2.21}$	69.11$_{\pm1.20}$	55.79$_{\pm4.27}$	79.69$_{\pm1.18}$	57.85$_{\pm2.34}$	74.70$_{\pm1.29}$	76.68$_{\pm1.56}$	**82.39$_{\pm1.29}$**
	Ovarian	75.05$_{\pm4.51}$	11.61$_{\pm1.25}$	75.55$_{\pm8.09}$	47.67$_{\pm8.33}$	28.02$_{\pm5.07}$	78.78$_{\pm10.51}$	15.58$_{\pm5.53}$	29.90$_{\pm14.62}$	47.63$_{\pm11.07}$	**91.83$_{\pm4.25}$**
	Gastric	65.11$_{\pm1.80}$	34.78$_{\pm6.32}$	64.53$_{\pm2.39}$	62.29$_{\pm3.50}$	NaN	34.65$_{\pm6.91}$	27.67$_{\pm2.85}$	62.30$_{\pm3.53}$	52.14$_{\pm9.50}$	**76.87$_{\pm3.09}$**
	Prostate	56.87$_{\pm4.38}$	53.10$_{\pm7.08}$	57.35$_{\pm4.49}$	64.71$_{\pm3.40}$	NaN	53.54$_{\pm6.20}$	51.05$_{\pm7.40}$	52.52$_{\pm7.85}$	56.62$_{\pm4.71}$	**75.25$_{\pm4.21}$**
	Gastric-Endo.	46.96$_{\pm6.41}$	36.16$_{\pm4.99}$	44.85$_{\pm12.37}$	50.26$_{\pm3.14}$	NaN	37.73$_{\pm4.77}$	34.92$_{\pm4.89}$	48.50$_{\pm2.26}$	49.80$_{\pm2.94}$	**65.93$_{\pm3.52}$**
Conch	Breast	97.22$_{\pm1.13}$	90.42$_{\pm4.39}$	96.94$_{\pm1.36}$	96.39$_{\pm1.60}$	89.04$_{\pm3.77}$	97.52$_{\pm0.87}$	90.49$_{\pm2.36}$	94.65$_{\pm0.43}$	95.99$_{\pm0.93}$	**97.48$_{\pm1.09}$**
	Lung	80.74$_{\pm3.87}$	43.45$_{\pm2.69}$	79.10$_{\pm5.12}$	67.97$_{\pm1.39}$	66.23$_{\pm3.97}$	79.10$_{\pm4.58}$	44.74$_{\pm1.81}$	66.55$_{\pm2.21}$	68.91$_{\pm2.45}$	**86.19$_{\pm1.20}$**
	Renal-3	83.83$_{\pm2.53}$	45.91$_{\pm1.94}$	84.37$_{\pm4.22}$	72.82$_{\pm2.70}$	69.92$_{\pm5.43}$	81.18$_{\pm3.83}$	46.27$_{\pm3.55}$	80.08$_{\pm2.16}$	80.74$_{\pm2.05}$	**87.59$_{\pm2.43}$**
	Renal-4	82.69$_{\pm1.43}$	47.35$_{\pm2.92}$	82.54$_{\pm1.95}$	74.68$_{\pm2.65}$	61.04$_{\pm12.52}$	81.28$_{\pm4.77}$	44.79$_{\pm1.91}$	76.32$_{\pm0.75}$	77.80$_{\pm1.08}$	**88.29$_{\pm1.35}$**
	Ovarian	88.06$_{\pm2.21}$	10.00$_{\pm1.10}$	86.69$_{\pm1.46}$	77.27$_{\pm3.23}$	24.60$_{\pm9.54}$	84.80$_{\pm3.67}$	11.06$_{\pm3.62}$	53.06$_{\pm10.45}$	59.64$_{\pm5.95}$	**92.98$_{\pm2.17}$**
	Gastric	79.28$_{\pm2.70}$	36.61$_{\pm5.97}$	79.78$_{\pm2.23}$	68.64$_{\pm2.41}$	NaN	56.54$_{\pm8.29}$	34.01$_{\pm3.36}$	63.33$_{\pm5.68}$	67.15$_{\pm4.62}$	**85.54$_{\pm0.91}$**
	Prostate	72.66$_{\pm2.76}$	53.75$_{\pm6.66}$	71.48$_{\pm4.19}$	70.54$_{\pm3.16}$	NaN	61.70$_{\pm10.36}$	55.23$_{\pm5.35}$	64.18$_{\pm2.19}$	66.90$_{\pm2.14}$	**79.87$_{\pm4.20}$**
	Gastric-Endo.	65.26$_{\pm6.18}$	36.76$_{\pm4.80}$	65.89$_{\pm4.81}$	56.65$_{\pm4.72}$	NaN	52.90$_{\pm9.44}$	37.66$_{\pm3.34}$	54.27$_{\pm2.30}$	52.31$_{\pm8.21}$	**73.16$_{\pm1.86}$**

Supplementary Table 7: The comparison results of spatial quantification (accuracy, presented in %). The best results are highlighted in bold.

RAMIL	WSI-AUC	Spatial-AUC	Spatial-ACC	Spatial-P	Spatial-R	Spatial-F1
Base	99.70$_{\pm0.16}$	89.22$_{\pm2.39}$	83.83$_{\pm2.53}$	88.33$_{\pm3.34}$	81.27$_{\pm2.61}$	84.64$_{\pm2.79}$
InD	**99.72$_{\pm0.11}$**	89.06$_{\pm2.20}$	82.70$_{\pm2.71}$	88.15$_{\pm2.13}$	79.08$_{\pm4.68}$	83.33$_{\pm3.33}$
InS	99.58$_{\pm0.16}$	**91.89$_{\pm1.53}$**	**87.30$_{\pm2.36}$**	**90.05$_{\pm2.86}$**	**86.39$_{\pm3.01}$**	**88.16$_{\pm2.54}$**
InD+InS	99.56$_{\pm0.18}$	90.63$_{\pm2.52}$	85.01$_{\pm3.93}$	89.07$_{\pm2.95}$	82.87$_{\pm6.07}$	85.78$_{\pm4.15}$
InD+InS+InR	99.57$_{\pm0.15}$	88.31$_{\pm2.19}$	82.83$_{\pm2.41}$	88.82$_{\pm2.89}$	78.53$_{\pm3.24}$	83.36$_{\pm2.96}$

Supplementary Table 8: Cross-model ablation results on RAMIL using the Renal-3 (TCGA-RCC) dataset, presented in %, The best results are highlighted in bold. ACC, P, R, F1 denote accuracy, precision, recall, and F1-score, respectively.

Variable	Description
$f(\cdot)$	Linear projection function
$g_c(\cdot)$	Attention network for category c
$\sigma(l)$	Output activation function (Sigmoid)
P_c	Bag-level prediction score for category c
L_c	Bag-level prediction logit for category c
$t(l)$	Tangent line to $\sigma(l)$ at L_c
L_{int}	x-axis value of the additional intersection point between $t(l)$ and $\sigma(l)$
l_i^c	Prediction logit (output of $f(\cdot)$) for instance i in category c
z_i^c	Raw attention score (output of $g_c(\cdot)$) for instance i in category c
a_i^c	Softmax-normalized attention score for instance i in category c

Supplementary Table 9: Description of Key Variables (Theorems)

Method	GFLOPs	Parameters	Memory	Inference time	Training time
SMMILe	1.50	1.20M	18.30MB	2.04s	25.68s, 26.71s
ABMIL	0.71	0.79M	12.11MB	1.34s	10.22s
TransMIL	2.70	2.67M	42.22MB	4.62s	17.82s

Supplementary Table 10: Computational cost analysis of MIL methods

1 Proofs

1.1 Proof of Theorem 1

Proof of Theorem 1. Revisiting $f(\cdot)$, typically designed as a linear projection involving two trainable vectors, denoted as $\mathbf{w}$ and $\mathbf{b}$. The attention scores are generated by $g(\cdot)$ with softmax normalization over the instances, thereby ensuring that $\sum_{k=1}^{K} a_k = 1$. Expanding Eq. (2), we derive:

$$\begin{aligned}
\mathbf{P} &= \sigma\left(f\left(\sum_{k=1}^{K} a_k * \mathbf{h}_k\right)\right) \\
&= \sigma\left(\mathbf{w}_f\left(\sum_{k=1}^{K} a_k * \mathbf{h}_k\right) + \mathbf{b}_f\right) \\
&= \sigma\left(\sum_{k=1}^{K} a_k * \mathbf{w}_f \mathbf{h}_k + \sum_{k=1}^{K} a_k * \mathbf{b}_f\right) \\
&= \sigma\left(a_1(\mathbf{w}_f \mathbf{h}_1 + \mathbf{b}_f) + \ldots + a_K(\mathbf{w}_f \mathbf{h}_K + \mathbf{b}_f)\right) \\
&= \sigma\left(\sum_{k=1}^{K} a_k * f(\mathbf{h}_k)\right).
\end{aligned} \tag{1}$$

where the bag-level logit is aggregated as $L = \sum_{k=1}^{K} a_k * l_k$, $l_k = f(\mathbf{h}_k)$. The resultant form represents a unique variant of IAMIL, which aggregates the raw outputs (logits) of $f(\cdot)$ followed by the activation function $\sigma(\cdot)$. □

1.2 Proof of Theorem 2

Proof of Theorem 2. Utilizing the chain rule of differentiation, the partial derivative of $\check{\mathcal{L}}_c$ with respect to $\check{\mathbf{w}}_g^c$ is formulated as:

$$\frac{\partial \check{\mathcal{L}}_c}{\partial \check{\mathbf{w}}_g^c} = \frac{\partial \check{\mathcal{L}}_c}{\partial \check{P}_c} * \frac{\partial \check{P}_c}{\partial \check{z}_i^c} * \frac{\partial \check{z}_i^c}{\partial \check{\mathbf{w}}_g^c}, \tag{2}$$

also, the partial derivative of $\hat{\mathcal{L}}_c$ with respect to $\hat{\mathbf{w}}_g^c$ is described as:

$$\frac{\partial \hat{\mathcal{L}}_c}{\partial \hat{\mathbf{w}}_g^c} = \frac{\partial \hat{\mathcal{L}}_c}{\partial \hat{P}_c} * \frac{\partial \hat{P}_c}{\partial \hat{z}_i^c} * \frac{\partial \hat{z}_i^c}{\partial \hat{\mathbf{w}}_g^c}, \tag{3}$$

Moreover, the derivative of $\check{P}_c$ in relation to $\check{z}_i^c$ is computed as:

$$\begin{aligned}
\frac{\partial \check{P}_c}{\partial \check{z}_i^c} &= \frac{\partial \sigma(\check{L}_c)}{\partial \check{L}_c} * \frac{\check{l}_i^c e^{\check{z}_i^c} \sum_{j=1}^{K} e^{\check{z}_j^c} - e^{\check{z}_i^c} \sum_{k=1}^{K} e^{\check{z}_k^c} \check{l}_j^c}{(\sum_{j=1}^{K} e^{\check{z}_j^c})^2} \\
&= \frac{\partial \sigma(\check{L}_c)}{\partial \check{L}_c} * \frac{e^{\check{z}_i^c}(\check{l}_i^c - \check{L}_c)}{\sum_{j=1}^{K} e^{\check{z}_j^c}},
\end{aligned} \tag{4}$$

and the derivative of $\hat{P}_c$ with respect to $\hat{z}_i^c$ is calculated as:

$$
\begin{aligned}
\frac{\partial \hat{P}_c}{\partial \hat{z}_i^c} &= \frac{e^{\hat{z}_i^c} \frac{1}{1+e^{-\hat{l}_i^c}} \sum_{j=1}^{K} e^{\hat{z}_j^c} - e^{\hat{z}_i^c} \sum_{k=1}^{K} e^{\hat{z}_k^c} * \frac{1}{1+e^{-\hat{l}_k^c}}}{(\sum_{j=1}^{K} e^{\hat{z}_j^c})^2} \\
&= \frac{e^{\hat{z}_i^c}(\hat{p}_c^i - \hat{P}_c)}{\sum_{j=1}^{K} e^{\hat{z}_j^c}},
\end{aligned} \tag{5}
$$

In light of Assumption 2, we deduce:

$$
\frac{\partial \check{\mathcal{L}}_c}{\partial \check{P}_c} * \frac{\partial \check{P}_c}{\partial \check{z}_i^c} * \frac{\partial \check{z}_i^c}{\partial \check{\mathbf{w}}_g^c} = \frac{\partial \hat{\mathcal{L}}_c}{\partial \hat{P}_c} * \frac{\partial \hat{P}_c}{\partial \hat{z}_i^c} * \frac{\partial \hat{z}_i^c}{\partial \hat{\mathbf{w}}_g^c}, \tag{6}
$$

where $\partial \check{\mathcal{L}}_c / \partial \check{P}_c = \partial \hat{\mathcal{L}}_c / \partial \hat{P}_c$. Furthermore, it can be readily demonstrated that $\partial \check{z}_i^c / \partial \check{\mathbf{w}}_g^c$ is equivalent to $\partial \hat{z}_i^c / \partial \hat{\mathbf{w}}_g^c$. This equivalence arises because the values of these two partial derivatives are exclusively dependent on the architecture of the attention networks and the input vector $\mathbf{h}_i$ (with $\check{z}_i^c = \check{g}_c(\mathbf{h}_i)$ and $\hat{z}_i^c = \hat{g}_c(\mathbf{h}_i)$), which remain consistent across RAMIL and IAMIL. Consequently, we can infer that:

$$
\begin{aligned}
\frac{\partial \check{P}_c}{\partial \check{z}_i^c} &= \frac{\partial \hat{P}_c}{\partial \hat{z}_i^c} \\
\frac{\partial \sigma(\check{L}_c)}{\partial \check{L}_c} * \frac{e^{\check{z}_i^c}(\check{l}_i^c - \check{L}_c)}{\sum_{j=1}^{K} e^{\check{z}_j^c}} &= \frac{e^{\hat{z}_i^c}(\hat{p}_c^i - \hat{P}_c)}{\sum_{j=1}^{K} e^{\hat{z}_j^c}} \\
\frac{e^{\check{z}_i^c}}{\sum_{j=1}^{K} e^{\check{z}_j^c}} / \frac{e^{\hat{z}_i^c}}{\sum_{j=1}^{K} e^{\hat{z}_j^c}} &= \frac{\hat{P}_c - \hat{p}_c^i}{\frac{\partial \sigma(\check{L}_c)}{\partial \check{L}_c}(\check{L}_c - \check{l}_i^c)} \\
\frac{\check{a}_i^c}{\hat{a}_i^c} &= \frac{\sigma(\hat{L}_c) - \sigma(\hat{l}_i^c)}{\frac{\partial \sigma(\check{L}_c)}{\partial \check{L}_c}(\check{L}_c - \check{l}_i^c)}.
\end{aligned} \tag{7}
$$

□

1.3 Proof of Theorem 3

Proof of Theorem 3. Firstly, The tangent line $t(l)$ of $\sigma(l)$ at L_c can be mathematically expressed as:

$$
t(l) = \frac{e^{-L_c} l}{(1 + e^{-L_c})^2} + \frac{1 + e^{-L_c} - L_c e^{-L_c}}{(1 + e^{-L_c})^2}, \tag{8}
$$

Given that the sigmoid function $\sigma(l)$ has a single inflection point at $l = 0$, and asymptotic property towards positive and negative infinity, it can be inferred that the tangent line $t(l)$ intersects with $\sigma(l)$ at an additional point, denoted as $(L_{int}, \sigma(L_{int}))$, where $t(L_{int}) = \sigma(L_{int})$. Importantly, the product of L_c and L_{int} is negative, *i.e.*, $L_c \cdot L_{int} < 0$. The sole exception to this rule occurs when $L_c = 0$, in which case there is only one intersection point, $L_c = L_{int} = 0$.

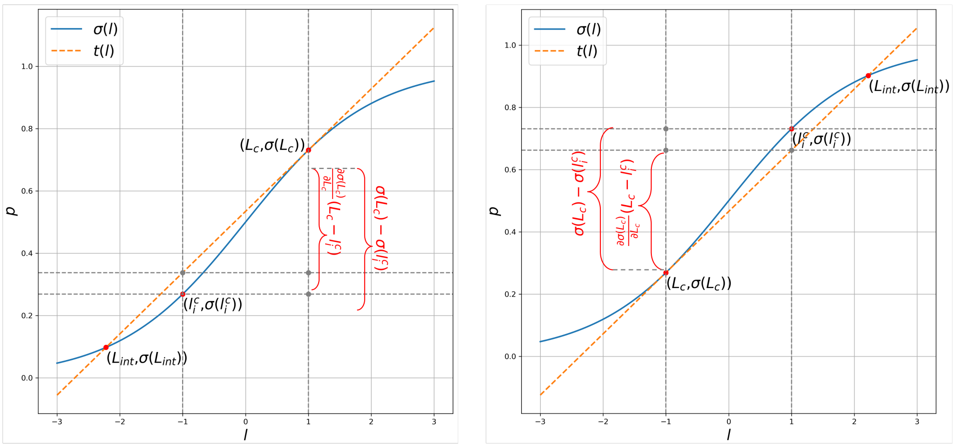

(a) (b)

Supplementary Note Fig. 1: In conditions where L_c is (a) greater than 0, and (b) less than 0, the geometric proof regarding Theorem 3 is presented.

Moreover, it can be readily demonstrated using the geometric approach (**Suppplementary Note Fig. 1**) that if $l_i^c \in (\min(L_c, L_{int}), \max(L_c, L_{int}))$, then $|\sigma(L_c) - \sigma(l_i^c)| > |\frac{\partial \sigma(\check{L}_c)}{\partial \check{L}_c}(\check{L}_c - \check{l}_i^c)|$, and vice versa.

Secondly, the intersection point L_{int} can be expressed as:

$$\begin{aligned} t(L_{int}) &= \sigma(L_{int}) \\ L_{int} &= \sigma(L_{int})(e^{-L_c} + e^{L_c} + 2) - e^{L_c} + L_c - 1, \end{aligned} \tag{9}$$

which is related to L_c. However, it does not have an analytical solution. Alternatively, we analyze the properties of Eq. (9) and give a series of discrete solutions using Newton's method. The first-order derivative of Eq. (9) with respect to L_c can be expressed as:

$$\frac{\partial L_{int}}{\partial L_c} = \sigma(L_{int})(e^{L_c} - e^{-L_c}) - e^{L_c} + 1, \tag{10}$$

with Taylor expansion of e^{L_c} and e^{-L_c}, we have:

$$\begin{aligned} \frac{\partial L_{int}}{\partial L_c} &= 2\sigma(L_{int}) \sum_{n=0}^{\infty} \frac{(L_c)^{2n+1}}{(2n+1)!} - \sum_{n=0}^{\infty} \frac{(L_c)^n}{n!} + 1 \\ &= (2\sigma(L_{int}) - 1) \sum_{n=0}^{\infty} \frac{(L_c)^{2n+1}}{(2n+1)!} - \sum_{n=1}^{\infty} \frac{(L_c)^{2n}}{2n!}, \end{aligned} \tag{11}$$

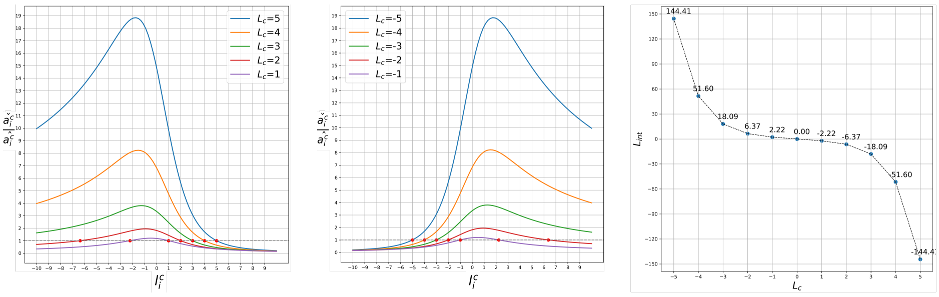

(a) (b) (c)

Supplementary Note Fig. 2: (a) Estimation results of L_{int} using Newton's method with different L_c. The ratio of $\check{a}_i^c$ to $\hat{a}_i^c$ with different l_i^c and L_c, (b) when $L_c > 0$, (c) when $L_c < 0$.

The second-order derivative of Eq. (9) with respect to L_c can be expressed as:

$$\frac{\partial^2 L_{int}}{\partial^2 L_c} = \sigma(L_{int})(e^{L_c} + e^{-L_c}) - e^{L_c}, \tag{12}$$

with Taylor expansion of e^{L_c} and e^{-L_c}, we have:

$$\begin{aligned} \frac{\partial^2 L_{int}}{\partial^2 L_c} &= 2\sigma(L_{int}) \sum_{n=0}^{\infty} \frac{(L_c)^{2n}}{2n!} - \sum_{n=0}^{\infty} \frac{(L_c)^n}{n!} \\ &= (2\sigma(L_{int}) - 1) \sum_{n=0}^{\infty} \frac{(L_c)^{2n}}{2n!} - \sum_{n=0}^{\infty} \frac{(L_c)^{2n+1}}{(2n+1)!}. \end{aligned} \tag{13}$$

Since $L_c \cdot L_{int} < 0$, if $L_c > 0$, it follows that $(2\sigma(L_{int}) - 1) < 0$, indicating that $\partial L_{int}/\partial L_c < 0$ and $\partial^2 L_{int}/\partial^2 L_c < 0$. Similarly, if $L_c < 0$, the first-order derivative $\partial L_{int}/\partial L_c$ is still less than 0, but the second-order derivative $\partial^2 L_{int}/\partial^2 L_c > 0$. If and only if $L_c = 0$, $\partial L_{int}/\partial L_c$ and $\partial^2 L_{int}/\partial^2 L_c$ are equal to 0. These properties of Eq. (9) indicate that (1) the distance between L_c and L_{int} increases as the absolute value of L_c increases, and (2) the rate of change for the absolute value of L_{int} also increases as the absolute value of L_c increases.

To elucidate the relationship between L_c and L_{int} more explicitly, we employed Newton's method to solve Eq. (9). For $L_c = \{0, 1, 2, 3, 4, 5\}$, the corresponding L_{int} values are $\{0.00, -2.22, -6.37, -18.09, -51.60, -144.41\}$, respectively. For $L_c < 0$, the L_{int} values are the negatives of their respective positive counterparts (**Supplementary Note Fig. 2c**). The results reveal that as L_c varies, the distance between L_c and L_{int} changes at an almost exponential rate.

Furthermore, we calculated the ratio of $\check{a}_i^c$ to $\hat{a}_i^c$ across different l_i^c and L_c values (**Supplementary Note Fig. 2a,b**). It can be seen that when the absolute value of L_c is relatively

large, the ratio of $\check{a_i^c}$ to $\hat{a_i^c}$ significantly exceeds 1 in most intervals, highlighting a substantial divergence between RAMIL and IAMIL in terms of attention scores allocated for low-discriminative and non-discriminative instances.

Considering the properties of the additional intersection point L_{int}, along with the expected convergence of $\sigma(L_c)$ towards either 0 or 1, a relatively large magnitude of $|L_c|$ is often observed, typically exceeding 5 in most cases (**Extended Data Fig. 1a**). Consequently, this results in $|L_{int}|$ being greater than 100. It becomes evident that satisfying the condition $|l_i^c| > |L_{int}|$ and $L_{int} \cdot l_i^c > 0$ is improbable. Moreover, for the majority of instances that meet the condition $l_i^c \in (\min(L_c, L_{int}), \max(L_c, L_{int}))$, the value of $\check{a_i^c}$ is expected to be several times greater than that of $\hat{a_i^c}$. □

Although these proofs have been proven using the sigmoid function, their applicability extends universally to the softmax function. Under the assumption that the raw outputs of other categories remain constant, and focusing solely on the relationship between the attention score a_k^c and the prediction score P_c for a single category, the softmax function can be effectively simplified to $1/(1 + \text{cst} \cdot e^{-L_c})$, where "cst" represents a constant. Notably, this simplified representation of the softmax function exhibits properties analogous to those of the sigmoid function.

2 Synthetic Experiment

In Theorem 3 and its proof, we assert the near improbability of the condition $|l_i^c| > |L_{int}|$ and $L_{int} \cdot l_i^c > 0$. This assertion plays a critical role in delineating the differences in attention score allocation between RAMIL and IAMIL. Consequently, to empirically observe the distributions of L_c, and l_i^c in both RAMIL and IAMIL, as well as to examine the distributions of the raw and softmax normalized attention scores (z_i^c, and a_i^c), we construct a synthetic dataset that follows the MIL setting (**Supplementary Table 1**).

For this synthetic MIL dataset, we generate a set of bags with corresponding bag-level labels where each bag consists of K instances, denoted as $\tilde{\mathbf{X}} = \{\tilde{\mathbf{x}}_1, \tilde{\mathbf{x}}_2, ..., \tilde{\mathbf{x}}_K\}$. Each instance is a vector with d dimensional features, $\tilde{\mathbf{x}}_k \in \mathbb{R}^d$. To simplify, this synthetic MIL dataset is defined with two categories. Consequently, we establish three types of instance distributions, used to generate discriminative (positive) instances for two categories, and non-discriminative (negative) instances. Each instance distribution is comprised of Gaussian distributions with different means and variances for each of the d dimensions, from which we sample each feature of instances. Therefore, the bag of each category is composed of a random proportion r of positive instances belonging to that category, supplemented with the remaining negative instances. It is important

to note that the positive instance distributions for the two categories are significantly different, while their distributions, compared to the negative instances, are similar but non-overlapping. This setup is designed to synthesize positive instances with high and low discriminative features. Ultimately, the dataset comprises a total of 2,00 bags, each containing 1,000 instances. The ratio of positive instances within each bag is randomly sampled from a range of 0.1 to 1. The dataset is evenly distributed across the two categories, with each category consisting of 100 bags.

For model training, we utilize the architecture of AB-MIL as the basis for RAMIL and modify it for IAMIL by conducting attention pooling at the instance level. Given that the instance $\tilde{\mathbf{x}}_k$ is not an image, we omit the embedding step. Only the linear projection function $f(\cdot)$, with 4 hidden nodes, and the gated attention network $g(\cdot)$, with 8 hidden nodes in the first layer and 4 hidden nodes in the second layer, are used and trained. The loss function employed is binary cross entropy computed for each category. The Adam optimizer is used, with the learning rate set to 0.001. To ensure a consistent level of convergence between RAMIL and IAMIL, the training process is stopped when the training loss does not decrease for 10 consecutive epochs.

Since there are only two categories of positive bags and no negative bags, the bag-level prediction logits L_{int} are always greater than 0. Thus, we present the experimental data for both categories in a unified manner (**Extended Data Fig. 1**). We observe that when either IAMIL or RAMIL converges, L_c values exceed 5 in most cases (**Extended Data Fig. 1(a)**), leading to L_{int} values dropping below -144. Thus, L_c and L_{int} define a relatively large interval. For both IAMIL and RAMIL, the majority of negative instances have l_i^c values lower than L_c (**Extended Data Fig. 1(b)**). Conversely, a small fraction of positive instances exhibit l_i^c values greater than L_c (**Extended Data Fig. 1(c)**). This confirms that a subset of positive instances with highly discriminative features has relatively high l_i^c values in the MIL process, exceeding the interval defined by L_c and L_{int}. Meanwhile, most negative instances have lower l_i^c values, positioning them within this interval. Following Theorem 3, this indicates that IAMIL assigns lower attention scores to most negative instances, while a subset of positive instances receives higher attention scores compared to those assigned by RAMIL.

This distinction is also evident from the distribution differences of raw (z_i^c) and softmax-normalized (a_i^c) attention scores in RAMIL and IAMIL. For positive instances, both distributions of raw (z_i^c) and softmax-normalized (a_i^c) attention scores exhibit a more skewed pattern compared to RAMIL, with a small subset of positive instances receiving significantly higher attention scores (illustrated by the extended orange tail in **Extended Data Fig. 1(d), (e)**). Conversely, for negative instances, both distributions of raw (z_i^c) and softmax-normalized (a_i^c) attention

scores in IAMIL are noticeably shifted towards lower values compared to RAMIL. (illustrated the distribution more concentrated towards the left on the x-axis, **Extended Data Fig. 1(f), (g)**).

3 Model Description

Reiterating the definition in the main manuscript, a set of patches $\{\mathbf{x}_1, \mathbf{x}_2, \ldots, \mathbf{x}_K\}$, each of size $D \times D$, are extracted from a WSI $\mathbf{X}$ of size $W \times H$, with the corresponding label Y. The objective of SMMILe is to learn a transformation function that maps the set of patches $\{\mathbf{x}_1, \mathbf{x}_2, \ldots, \mathbf{x}_K\}$ to the WSI-level label $\mathbf{Y}$. Concurrently, it also aims to predict instance-level labels $y_1, y_2, \ldots, y_K$ for each individual patch.

In MIL settings, supervision is exclusively available at the WSI level. Previous research has predominantly focused on binary or multi-class classification, where each WSI is assigned to a single category, *i.e.*, $\mathbf{Y}$ is represented as a scalar in binary classification or as a C-dimensional one-hot vector in multi-class classification. In this paper, we expand SMMILe to accommodate multi-label classification. Consequently, $\mathbf{Y} = \{Y_1, Y_2, \ldots, Y_C\}$ is configured as a C-dimensional vector, with each element Y_c being a binary indicator that is independently distributed, representing the presence or absence of each category in this WSI. This adaption aligns SMMILe with more general pathology scenarios, capturing multiple phenotypic categories that may concurrently exist in a single WSI.

3.1 Network Architecture

The proposed network architecture begins with a pretrained encoder $e(\cdot)$, mapping all instances $\{\mathbf{x}_1, \mathbf{x}_2, \ldots, \mathbf{x}_K\}$ to a uniform embedding space $\{\mathbf{h}_1, \mathbf{h}_2, \ldots, \mathbf{h}_K\}$. Given the large number of instances contained within each WSI, the encoder training becomes time-consuming and computationally strenuous, leading to the parameters of the encoder generally being kept frozen. Taking ResNet-50 as the encoder $e(\cdot)$ example. Feature maps are extracted following the third residual block and aggregated through global average pooling, producing a 1024-dimensional embedding for each instance.

Subsequently, a convolutional layer $cov(\cdot)$ is introduced, which further maps the instance embeddings to a lower dimension. The parameters of $cov(\cdot)$ are trainable for increasing the flexibility of the entire MIL framework. While existing works often employ a linear projection layer for this function, we replace it with a convolutional layer to enhance the representation ability of our framework for downstream tasks by introducing WSI-level local receptive fields. Similar to NIC, we reposition the instance embeddings $\{\mathbf{h}_1, \mathbf{h}_2, \ldots, \mathbf{h}_K\}$ according to their positions in the WSI, and fill other positions with zero embeddings, creating a compressed WSI $\mathbf{H}_{nic} \in \mathbb{R}^{\frac{W}{D} \times \frac{H}{D} \times 1024}$

to enable the convolution operation. We utilize 128 convolutional kernels with size 3×3 and padding operation, ensuring that the size of the compressed WSI is maintained after convolution, *i.e.*, $\mathbf{H}'_{nic} = cov(\mathbf{H}_{nic})$, where $\mathbf{H}'_{nic} \in \mathbb{R}^{\frac{W}{D} \times \frac{H}{D} \times 128}$. Then, the compressed embeddings $\{\mathbf{h}'_1, \mathbf{h}'_2, \ldots, \mathbf{h}'_K\}$ of all instances can be obtained from $\mathbf{H}^{'}_{nic}$ based on their respective positions. It is important to note that when the size of convolutional kernels is set to 1×1, $cov(\cdot)$ degrades to a linear projection layer, which is particularly effective for specific MIL tasks with very limited positive instances in each bag, such as cancer metastasis detection.

In the final stage, the compressed embedding of each instance $\mathbf{h}'_k$, serves as the input for an instance detector $g(\cdot)$ and an instance classifier $f(\cdot)$. The instance detector, analogous to the attention network in RAMIL frameworks, utilizes the gated attention mechanism. It comprises three linear projection layers with 64, 64, and C hidden nodes, respectively, assigning category-wised raw attention scores $\{z^1_k, z^2_k, \ldots, z^C_k\}$ to each instance. Then these raw attention scores $\{z^c_1, z^c_2, \ldots, z^c_K\}$ of each category are normalized via softmax function across all instances, resulting in detection (attention) scores $\{a^c_1, a^c_2, \ldots, a^c_K\}$, where the sum of them remains invariant to K and equals to 1. The instance classifier $f(\cdot)$ is a linear projection layer with C hidden nodes. It maps the compressed embedding $\mathbf{h}'_k$ of each instance to category-related scalars $\{l^1_k, l^2_k, \ldots, l^C_k\}$. These scalars are then normalized over categories using the softmax function for multi-class classification, or the sigmoid function for binary and multi-label classification, yielding the classification scores $\{p^1_k, p^2_k, \ldots, p^C_k\}$ for each instance.

The bag-level prediction score P_c for each category is then obtained by taking the dot product of classification and detection scores, and summing them up.

3.2 Instance-based Comprehensive Attention

To enhance the comprehensive attention capability of SMMILe toward all discriminative instances, we adhere to the traditional MIL by categorizing bags into two types: negative bags and positive bags. Negative bags do not contain any positive instances of any category, as exemplified by WSI of normal tissue; whereas positive bags include positive instances of one or several categories. We propose (1) an attention consistency constraint for negative bags; (2) a parameter-free instance dropout module; and (3) a superpatches-based delocalised instance sampling module for positive bags. It is worth noting that the instance dropout and instance sampling modules presented in this section introduce diversity in instance combinations of each bag, effectively serving as two forms of bag-level augmentation. This, in turn, enhances the performance of bag-level predictions.

Consistency Constraint

A bag is classified as negative only if none of the instances within it belong to a positive category. Thus, the classification of negative bags should not rely on a subset of instances but rather on all instances. Instead of applying the same attention mechanism to both negative and positive bags like previous RAMIL approaches, we introduce a consistency constraint for the attention mechanism, which restricts all instances in a negative bag should having the same attention score. This consistency loss is defined as follows:

$$\mathcal{L}_{cons} = \frac{1}{CK} \sum_{c=1}^{C} \sum_{k=1}^{K} \left(a_k^c - \bar{a}^c\right)^2, \tag{14}$$

where $\bar{a}^c = \frac{1}{K} \sum_{k=1}^{K} a_k^c$. By applying this MSE loss penalty, the attention scores across all instances in a negative bag become uniform, effectively ensuring that no individual instance makes special contributions to any category. This uniformity explicitly boosts the classification accuracy for negative bags as well as the recognition ability of SMMILe to negative instances, thereby implicitly improving the comprehensive attention for discriminative instances.

Parameter-Free Instance Dropout

The comprehensive attention capability of SMMILe is limited by focusing mainly on high-discriminative instances and neglecting others. An intuitive idea is that during the training process, omitting high-discriminative instances while retaining bag-level supervision could encourage the model to focus on the remaining discriminative instances. This approach introduces an additional challenge: determining the ideal instance dropout rate. Specifically, excessive instance dropout during early, unstable training phases may obstruct model convergence. In contrast, a low dropout rate in stable phases offers limited benefits. Additionally, with substantial variation in positive instance proportions across different bags, selecting a uniform dropout rate effective for all bags is unfeasible.

To resolve this issue, we design a parameter-free instance dropout module. Instead of applying dropout to attention scores, which do not directly reflect the contribution of each instance towards the bag-level prediction score, this module targets the instance scores (*i.e.*, the product of classification and detection scores for each instance), which have strict marginal contributions to the bag-level prediction score. For a set of instance scores $\{I_1^c, I_2^c, \ldots, I_K^c\}$ of a bag, where $P_c = \sum_{k=1}^{K} I_k^c$, and $I_k^c = a_k^c \cdot p_k^c$, we first apply Min-Max normalization to obtain normalized instance scores $\{\breve{I}_1^c, \breve{I}_2^c, \ldots, \breve{I}_K^c\}$. Then, a corresponding set of random floating-point numbers $\{\eta_1^c, \eta_2^c, \ldots, \eta_K^c\}, \eta \in [0, 1]$ is generated. We compare them pairwise to obtain the instance drop masks $\{O_1^c, O_2^c, \ldots, O_K^c\}$. Finally, these instance drop masks are applied to the instance scores,

and the bag-level prediction score with instance dropout for category c is computed as:

$$P_c^{dp} = \sum_{k=1}^{K} O_k^c \cdot I_k^c, \tag{15}$$

where $O_k^c = [\breve{I}_k^c < \eta_k^c]$, $[\cdot]$ denoting an Iverson bracket. It can be observed that the proposed instance dropout module does not require any additional hyperparameters. The decision to drop an instance is based on its instance score I_k^c. The higher I_k^c, the less likely it is to meet the condition $\breve{I}_k^c < \eta_k^c$, making it more prone to be dropped.

Superpatch-based Delocalised Instance Sampling

Instance sampling emerges as another viable solution for enhancing the comprehensive attention capability of SMMILe. By performing multiple rounds of random sampling within a bag, each sampling generates a pseudo-bag composed of a subset of instances. The bag-level supervision is then applied to guide predictions for these pseudo-bags. This approach enables the model to focus on diverse sets of discriminative instances in each pseudo-bag, thus improving its comprehensive attention ability. Nevertheless, this kind of random sampling is uncontrollable and may lead to some pseudo-bags lacking positive instances, thereby introducing substantial noise into the training process of the model. Here, we propose a superpatch-based delocalised instance sampling module to address this issue. Recall the compressed WSI $\mathbf{H}_{nic}$ we constructed for a bag, wherein each pixel corresponds to a patch in the original WSI. We employ the Simple Linear Iterative Clustering (SLIC), a widely-utilized, non-trainable clustering-based partition algorithm, to generate a set of superpatches $\{\mathrm{SP}_1, \mathrm{SP}_2, \ldots, \mathrm{SP}_S\}$ from $\mathbf{H}_{nic}$, where S indicates the number of superpatches of each bag. This leads to patches that are spatially close with similar representations being grouped into the same superpatch. Based on these superpatches, instances of a bag can be divided into S subsets. By conducting T rounds of random sampling with replacement, where each round involves sampling one instance from each subset to create a pseudo-bag with S delocalised instances, a total of T pseudo-bags are generated. The instance sampling is also performed on instance scores directly. For t-th pseudo-bag, we have the sampled instance scores $\{\tilde{I}_{1,t}^c, \tilde{I}_{2,t}^c, \ldots, \tilde{I}_{S,t}^c\}$ for category c, where $\tilde{I}_s^c$ is an instance score sampled from superpatch SP_s, and the bag-level prediction score of t-th pseudo-bag is calculated as:

$$P_{c,t}^{sp} = \sum_{s=1}^{S} \tilde{I}_{s,t}^c, \tag{16}$$

Owing to the characteristic of superpatch, each pseudo-bag is composed of instances exhibiting varied spatial and representation distributions, providing the necessary diversity to encompass both positive and negative instances. Furthermore, sampling instances randomly from superpatches in each round results in a diverse array of instance combinations. This variety mitigates the overshadowing effect of high-discriminative instances on low-discriminative instances within the same pseudo-bag, consequently encouraging the model to focus more on those low-discriminative instances. Additionally, integrating instance sampling with instance dropout is particularly beneficial in scenarios where positive instances are predominant within a bag, such as subtyping on primary tumor slides. The bag-level prediction score for the t-th pseudo-bag, when instance dropout is applied, is computed as follows:

$$P_{c,t}^{sdp} = \sum_{s=1}^{S} O_s^c \cdot \tilde{I}_{s,t}^c, \tag{17}$$

where O_s^c is the instance drop mask for $\tilde{I}_s^c$. This can be regarded as masking high-discriminative superpatches.

Finally, all bag-level predictions generated by SMMILe, *i.e.*, P_c, P_c^{dp}, $\{P_{c,1}^{sp}, P_{c,2}^{sp}, \ldots, P_{c,T}^{sp}\}$, and $\{P_{c,1}^{sdp}, P_{c,2}^{sdp}, \ldots, P_{c,T}^{sdp}\}$, are supervised by bag-level label Y_c of each category. The classification loss for each bag is calculated as:

$$\begin{aligned} \mathcal{L}_{cls} = & \frac{1}{C} \sum_{c=1}^{C} \left(\mathrm{BCE}(P_c, Y_c) + \mathrm{BCE}(P_c^{dp}, Y_c) \right) \\ & + \frac{1}{CT} \sum_{t=1}^{T} \sum_{c=1}^{C} \left(\mathrm{BCE}(P_{c,t}^{sp}, Y_c) + \mathrm{BCE}(P_{c,t}^{sdp}, Y_c) \right), \end{aligned} \tag{18}$$

where $\mathrm{BCE}(P, Y)$ stands for the binary cross entropy loss between the prediction P and the true label Y.

3.3 MRF-based Instance Refinement

The aforementioned modules endow SMMILe with the capability to distinguish between positive and negative instances within each bag for different categories. However, due to the diversity among different bags, such as variations in feature distributions and significant differences in the proportions of positive instances, it is nearly impossible to choose a unified decision boundary for instance-level classification across varying bags and categories. Consequently, we design an instance refinement network to align features of instances of the same category within different

bags, thereby enabling us to learn a unified instance-level classification decision boundary across diverse bags.

Instance Refinement Network

The instance refinement network is structured with N linear layers $\{v_1(\cdot), v_2(\cdot), \ldots, v_N(\cdot)\}$. Each layer is implemented with $(C+1)$ hidden nodes and employs a softmax activation function for output generation. Here, C represents the number of categories for WSIs, excluding the negative category. For the negative WSI, the label $\mathbf{Y}$ is encoded using a C-dimensional vector of zeros. Consequently, the term $(C+1)$ incorporates the negative category to account for instances. These linear layers are assigned the identical task of generating predictions for individual instances but involve different sets of instances with associated compressed embeddings and pseudo-labels for training.

The challenge lies in acquiring pseudo-labels for network training. Leveraging the distinguishing capability of SMMILe for instances of different categories within each bag, we propose an online sample selection and labeling strategy. During each epoch of training, we can obtain a set of instance scores $\{I_1^c, I_2^c, \ldots, I_K^c\}$ for category c from SMMILe. From this set, we select the top θ percent of instances, labeling them as belonging to category c. This selection is restricted to the categories present in each bag. For negative samples, we first compute the mean score across different categories for each instance, represented as $\bar{I}_k = \frac{1}{C}\sum_{c=1}^{C} I_k^c$. Subsequently, we select the bottom θ percent of instances from these averages $\{\bar{I}_1, \bar{I}_2, \ldots, \bar{I}_K\}$, labeling them as negative, *i.e.*, category $(C+1)$. Consequently, the first linear layer $v_1(\cdot)$ is trained using the compressed embeddings and pseudo-labels of the selected instances, represented as $\{(\mathbf{h}'_1, \breve{y}_1^1), (\mathbf{h}'_2, \breve{y}_2^1), \ldots, (\mathbf{h}'_J, \breve{y}_J^1)\}$, where J denotes the total number of selected instances, and $\breve{y}_j^1$ is the first-round pseudo-label of j-th selected instance.

Building on the concept of self-training, we employ the prediction results of instances from $v_1(\cdot)$ to supervise the learning of $v_2(\cdot)$, and similarly for subsequent layers, to achieve a higher degree of instance refinement. Specifically, the compressed embeddings of all instances in a bag are fed into $v_n(\cdot)$, yielding prediction scores of these instances, denoted by $\{\mathbf{p}_1^n, \mathbf{p}_2^n, \ldots, \mathbf{p}_K^n\}$, where $\mathbf{p}_k^n = v_n(\mathbf{h}'_k)$ is a $(C+1)$-dimensional vector, where each element represents the probability of the instance belonging to a corresponding category. Then, the proposed online sample selection and labeling strategy is employed here, the selected instances and $(n+1)$-round pseudo-labels are used to train the subsequent linear layer $v_{(n+1)}(\cdot)$. It is crucial to note that since each linear layer in the instance refinement network can generate predictions for the negative category $(C+1)$, there is no requirement for a separate selection process for negative samples, except for the training

process of $v_1(\cdot)$. Also, the instances selected for each linear layer are likely to differ, while the total number J remains constant. Through this strategy, every linear layer in the instance refinement network can be concurrently trained with the SMMILe primary network in each epoch. The refinement loss is defined as:

$$\mathcal{L}_{ref} = \frac{1}{NJ} \sum_{n=1}^{N} \sum_{j=1}^{J} \mathrm{CE}\left(\mathbf{p}_j^n, \breve{y}_j^n\right), \tag{19}$$

where $\mathrm{CE}(\mathbf{p}, y)$ stands for the categorical cross entropy loss between the prediction $\mathbf{p}$ and the pseudo-label y. The optimization process of the instance refinement network also facilitates learning more uniform discriminative instance features (generated by $cov(\cdot)$) across different bags, which enhances the bag-level prediction performance of SMMILe.

Superpatch-based MRF Constraint

Nevertheless, the proposed sample selection strategy, which treats each instance as an independent entity and only high-scoring instances from each category can be used for supervision, may induce prediction biases in the instance refinement network. Moreover, the current instance refinement network ignores the spatial relationship between instances (patches) within a bag (WSI), which is important for the comprehensive detection of positive instances. Therefore, we introduce a superpatch-based MRF constraint that incorporates local spatial smoothness at the WSI level. This constraint requires the minimization of both the first-order energy within each superpatch and the second-order energy between adjacent superpatches. Consider the n-th linear layer $v_n(\cdot)$. The prediction scores for instances within the superpatch SP_s are represented as $\{\mathbf{p}_1^n, \mathbf{p}_2^n, \ldots, \mathbf{p}_{|\mathrm{SP}_s|}^n\}$, where $|\mathrm{SP}_s|$ signifies the count of instances in this superpatch. Furthermore, the prediction score for superpatch SP_s is calculated as $\bar{\mathbf{p}}_s^n = \frac{1}{|\mathrm{SP}_s|} \sum_{sp=1}^{|\mathrm{SP}_s|} \mathbf{p}_{sp}^n$, and it is surrounded by M_s adjacent superpatches, whose prediction scores are denoted by $\{\bar{\mathbf{p}}_{s,1}^n, \bar{\mathbf{p}}_{s,2}^n, \ldots, \bar{\mathbf{p}}_{s,M_s}^n\}$. The MRF constraint loss for superpatch SP_s, incorporating both first-order and second-order energy, is defined as follows:

$$\begin{aligned}\mathcal{L}_{mrf} = \frac{1}{N} \sum_{n=1}^{N} \Bigg(& \frac{\lambda_1}{|\mathrm{SP}_s|} \sum_{sp=1}^{|\mathrm{SP}_s|} \left\|\mathbf{p}_{sp}^n - \bar{\mathbf{p}}_s^n\right\|^2 \\ & + \frac{\lambda_2}{M_s} \sum_{m=1}^{M_s} \left\|\bar{\mathbf{p}}_{s,m}^n - \bar{\mathbf{p}}_s^n\right\|^2 \Bigg).\end{aligned} \tag{20}$$

where λ_1 and λ_2 control the balance between first-order and second-order energy. This constraint can implicitly propagate the pseudo-label information of high-scoring instances to the local regions

constrained by superpatches, thereby enhancing the spatial smoothness of instance prediction scores.

4 Dataset Description

Breast (Camelyon16), employed for metastasis detection in breast cancer, exemplifies a classic binary classification task in WSI analysis. It comprises 399 WSIs with or without metastasis, each accompanied by detailed pixel-level annotations.

Lung (TCGA-LU) employed for subtyping in non-small cell lung cancer comprises a total of 937 WSIs, categorizing them into two subtypes: adenocarcinoma (LUAD) and squamous cell carcinoma (LUSC). The cancerous region of 523 WSIs was entirely annotated at the pixel level by two experienced pathologists and four medical students.

Ovarian (UBC-OCEAN) employed for subtyping in ovarian cancer, comprises a total of 513 WSIs, categorizing them into five subtypes: high-grade serous cancerous (HGSC), low-grade serous cancerous (LGSC), endometrioid cancerous (EC), clear cell carcinoma (CC), and mucinous cancerous (MC). Part of the cancerous, healthy, or necrotic regions of 152 WSIs were annotated. We combined the healthy and necrotic annotations to categorize them as normal tissue.

RCC-3 (TCGA-RCC) includes a total of 660 WSIs, with three subtypes, clear cell RCC (CCRCC), papillary RCC (PRCC), and chromophobe RCC (CHRCC). The cancerous region of 338 WSIs was entirely annotated at the pixel level by two experienced pathologists and four medical students.

RCC-4 (IH-RCC) collected from the First Affiliated Hospital of Xi'an Jiaotong University, with ethical approval (KYLLSL2021-420). It encompasses 563 WSIs from 168 patients across four RCC subtypes, CCRCC, PRCC, CHRCC, and Renal Oncocytoma (ROCY), with approximately 40 patients per subtype. The cancerous region of 138 WSIs was entirely annotated at the pixel level.

Gastric Endoscopy (IH-ESD) collected from the First Affiliated Hospital of Xi'an Jiaotong University, with ethical approval (KYLLSL2022-333). It includes 99 WSIs with early gastric cancer Endoscopic Submucosal Dissection (ESD) specimens, a total of 286 tissue samples. Each tissue sample was meticulously annotated at the pixel level, with three categories, *i.e.*, tumor, inflammation, and normal tissue.

Gastric (TCGA-STAD), comprising 339 WSIs, was sourced from the TCGA Database. As the TCGA database does not provide detailed classification information for gastric adenocarcinoma, two pathologists with over a decade of experience classified all WSIs following the

World Health Organization (WHO) histological classification system. At the same time, they performed a detailed, patch-level annotation on 128 WSIs, identifying three tissue subtypes: highly differentiated (papillary and tubular), poorly differentiated, and mucinous.

Prostate (SICAPv2), a publicly available prostate Gleason grading dataset, includes 153 WSIs labeled with categories G3, G4, G5, and normal tissue. This dataset provides tessellated patches with corresponding coordinates. Although the majority of patches come with annotations, a notable subset is devoid of labels. Two experienced pathologists provided supplementary annotations for these unlabelled patches.

5 Implementation Information

Patch embeddings used for all methods are extracted from two backbone encoders: the third residual block of the ResNet-50, pretrained on the ImageNet dataset, and the final layer of the Conch model, pretrained on large-scale pathology datasets. Where possible, configurations were aligned with the original implementations or their corresponding publications. It is, however, important to underline that the majority of these baselines are not intrinsically designed to accommodate multi-label classification tasks. Thus, modifications were introduced to these methods, with a particular emphasis on enhancing the attention aggregation mechanism, thereby extending their functionality to support multi-label classification. There is an exception, TransMIL is based on the self-attention mechanism and cannot be modified to multi-class attention. Therefore, in the experiment, TransMIL is unable to output patch-level predictions in multi-label datasets. Also, a weighted sampling technique is incorporated during the sample selection phase for all baselines, including the proposed SMMILe, to mitigate the issue of class imbalance. Furthermore, except for SMMILe, which possesses an instance refinement network capable of directly generating patch-level predictions, the derivation of patch-level predictions in representation-based attention MIL baselines, such as RAMIL, CLAM, DSMIL, TransMIL, and DTFD-MIL, relies on the raw attention scores. In the case of NIC and NIC-WSS, patch-level predictions are acquired from grad-CAM outputs, whereas for IAMIL, AddMIL, and the variants of SMMILe without integrating with the instance refinement network, predictions are based on instance scores.

In the configuration of SMMILe, the kernel size of convolutional layer $cov(\cdot)$ is set to 1×1 for the Camelyon16 dataset, and 3×3 for other datasets. The super-patches are generated using the Simple Linear Iterative Clustering (SLIC) over-segmentation algorithm, as implemented in the scikit-image package. Considering both the high spatial homogeneity and the significant proportion of tumor areas in WSIs of multi-class classification datasets, the initial size of super-patches is set to 5×5 for multi-class datasets and 3×3 for others. Also, the integration of instance

sampling with dropout is specifically utilized for multi-class classification datasets. The total number of sampling rounds T is set to 10, and the control parameters for the MRF constraint, λ_1 and λ_2, are fixed at 0.8 and 0.2, respectively, for all datasets.

The training of SMMILe is divided into two stages. In the first stage, the primary network of SMMILe is trained using the consistency loss ($\mathcal{L}cons$) and the bag-level classification loss ($\mathcal{L}cls$), for up to 200 epochs with the ResNet-50 encoder and 40 epochs with the Conch encoder. In the second stage, the number of linear layers N in the instance refinement network is set to 3, with a sample selection rate θ of 10% for each category, adjusted to 1% for the Camelyon dataset due to the limited proportion of tumor regions. Both the primary and instance refinement networks are then trained using all loss functions, including $\mathcal{L}_{cons}$, $\mathcal{L}_{cls}$, $\mathcal{L}_{ref}$, and $\mathcal{L}_{mrf}$, for up to 100 epochs with the ResNet-50 encoder and 20 epochs with the Conch encoder. The Adam optimizer is used with a learning rate of $2e^{-5}$, and an early stopping strategy is implemented for both stages. For inference, the bag-level output score P_c for each category and the instance-level output scores $\{\mathbf{p}_1^N, \mathbf{p}_2^N, \ldots, \mathbf{p}_K^N\}$ from the last linear layer $v_N(\cdot)$ are employed as the prediction results for the WSI and the corresponding patches, respectively. For all experiments, we utilized patient-level or WSI-level 5-fold cross-validation to assess the predictive performance of each model. WSI-level splits were conducted only when patient information was unavailable, with all splits performed randomly. In each fold, 80% of the data was randomly split into a train-validation set (90% for training and 10% for validation), while the remaining 20% was used as a test set.

All experiments were conducted on an HPC system with NVIDIA A100 GPUs (40 GB), using a Python 3.10.4 environment (venv-based) configured with GCCcore 11.3.0, CUDA 11.3.1, and cuDNN 8.2.1.32. Core libraries included PyTorch 1.12.1 and TorchVision 0.13.1, with additional dependencies available in our GitHub repository (https://github.com/ZeyuGaoAi/SMMILe). OpenSlide 3.4.1 was used for whole-slide image processing.

6 Cross-model Ablation

To further explore the adaptability of SMMILe's modules across different MIL frameworks, we investigate their effects when integrated into RAMIL. Specifically, we examine how the key modules, such as *InD*, *InS*, and *InR* perform within RAMIL, originally derived from CLAM but with the instance clustering module removed. Previous experiments suggest that this module has minimal impact on spatial quantification.

For efficiency, we conducted cross-module ablation experiments on the Renal-3 (TCGA-RCC) dataset. The objective was to assess whether SMMILe's modules remain effective when applied to a fundamentally different MIL framework (RAMIL). While IAMIL aggregates at the score level,

RAMIL performs aggregation at the representation level, which, according to our theoretical findings, may affect the effectiveness of score-based operations, *i.e.*, dropout (*InD*) and pseudo-label generation (*InR*). Experimental results (**Supplementary Table 8**) align with expectations:

- ***InD* and *InR* are less effective in RAMIL** – Since RAMIL aggregates at the representation level rather than the score level, attention scores do not directly reflect their contribution to the final bag prediction. Consequently, the dropout mechanism in *InD* and the pseudo-label generation in *InR* are less accurate in this setting.
- ***InS* significantly improves spatial quantification in RAMIL** – Unlike *InD* and *InR*, the delocalized sampling strategy in *InS* does not rely on instance scores. This allows it to adapt well to RAMIL and potentially other representation-based MIL frameworks.

These results reinforce our rationale for selecting IAMIL as the foundation of SMMILe and highlight the significance of instance-based MIL in achieving precise spatial quantification. By enabling more accurate allocation of instance scores, instance-based MIL enhances interpretability and improves performance in computational pathology tasks.

7 Computational Cost

Under the specified experimental conditions—CUDA 11.3.1, Python 3.10.4, PyTorch 1.12.1—on an HPC system with A100 GPUs (40GB) and a patch embedding dimension of 1024, we performed a quantitative computational cost analysis. Compared to ABMIL and TransMIL—one classic and one transformer-based MIL model—SMMILe's computational cost (excluding training time) remains within the same order of magnitude as ABMIL, while being significantly lower than TransMIL. Specifically, SMMILe requires 1.50 GFLOPs and 1.20M parameters, which is higher than ABMIL (0.71 GFLOPs, 0.79M params) but lower than TransMIL (2.70 GFLOPs, 2.67M params). In terms of memory, SMMILe allocates 18.30 MB, compared to ABMIL's 12.11 MB and TransMIL's 42.22 MB. For inference, SMMILe takes approximately 1.5× longer per 100 WSIs than ABMIL (2.04s vs. 1.34s) but significantly less time than TransMIL (2.04s vs. 4.62s). However, when considering the full WSI processing pipeline—including patch tessellation (about 1s per WSI) and embedding extraction (ranging from 10s to 1 min per WSI) using a pretrained encoder—this additional cost becomes negligible in the overall workflow.

For training, SMMILe (Stage I) takes approximately 2.5× and 1.5× longer per epoch (1000 samples) than ABMIL and TransMIL, respectively (25.68s vs. 17.82s vs. 10.22s). However, most MIL models converge within 5 to 10 epochs when using patch embeddings from the pathology foundation model (Conch). For a dataset of 1,000 WSIs, SMMILe (Stage I) converges within 4

minutes, with an additional 2 minutes for Stage II, requiring half the number of epochs and an epoch time of 26.71s. In comparison, ABMIL and TransMIL complete training in approximately 1–2 minutes and 3 minutes, respectively. While this difference is notable, in the era of large-scale models, the additional computational cost—at the minute level—remains insignificant compared to the gains in performance and expressiveness. The primary contributor to SMMILe's higher training time is the Delocalised Instance Sampling (InS) module, which performs iterative sampling for each superpixel within each WSI during every epoch and accounts for approximately 60% of the total training time overhead. However, InS plays a crucial role in spatial quantification, significantly enhancing SMMILe's performance, and has potential applicability to other MIL frameworks. Future optimizations, such as multi-threading or preprocessing, could further improve its efficiency while preserving its spatial quantification benefits.
