# Supplementary material for: SMMILe enables accurate spatial quantification in digital pathology using multiple-instance learning
Source: Nat Cancer. 2025 Nov 19;6(12):2025–41. doi: 10.1038/s43018-025-01060-8 (PMC12727519; doi:10.1038/s43018-025-01060-8)
Supplement: Supplementary file 2 — Reporting Summary [file 43018_2025_1060_MOESM2_ESM.pdf]

nature portfolio

Corresponding author(s): Mireia Crispin-Ortuzar

Last updated by author(s): Jul 22, 2025

Reporting Summary

Nature Portfolio wishes to improve the reproducibility of the work that we publish. This form provides structure for consistency and transparency in reporting. For further information on Nature Portfolio policies, see our Editorial Policies and the Editorial Policy Checklist.

Statistics

For all statistical analyses, confirm that the following items are present in the figure legend, table legend, main text, or Methods section.

n/a	Confirmed	
☐	☒	The exact sample size (n) for each experimental group/condition, given as a discrete number and unit of measurement
☐	☒	A statement on whether measurements were taken from distinct samples or whether the same sample was measured repeatedly
☒	☐	The statistical test(s) used AND whether they are one- or two-sided<br>*Only common tests should be described solely by name; describe more complex techniques in the Methods section.*
☒	☐	A description of all covariates tested
☒	☐	A description of any assumptions or corrections, such as tests of normality and adjustment for multiple comparisons
☐	☒	A full description of the statistical parameters including central tendency (e.g. means) or other basic estimates (e.g. regression coefficient) AND variation (e.g. standard deviation) or associated estimates of uncertainty (e.g. confidence intervals)
☒	☐	For null hypothesis testing, the test statistic (e.g. F, t, r) with confidence intervals, effect sizes, degrees of freedom and P value noted<br>*Give P values as exact values whenever suitable.*
☒	☐	For Bayesian analysis, information on the choice of priors and Markov chain Monte Carlo settings
☒	☐	For hierarchical and complex designs, identification of the appropriate level for tests and full reporting of outcomes
☒	☐	Estimates of effect sizes (e.g. Cohen's d, Pearson's r), indicating how they were calculated

Our web collection on statistics for biologists contains articles on many of the points above.

Software and code

Policy information about availability of computer code

Data collection	Whole Slide Images (WSIs) of IH-RCC and IH-ESD were collected using a KFBIO-005 digital slide scanner (Jiangfeng Biology) at 20× magnification (0.5µm/pixel resolution) at the First Affiliated Hospital of Xi'an Jiaotong University. Public datasets, including TCGA-LU (LUAD and LUSC), TCGA-RCC, and TCGA-STAD, were downloaded from the Genomic Data Commons portal [https://portal.gdc.cancer.gov] using the GDC Application Programming Interface [https://gdc.cancer.gov/developers/gdc-application-programming-interface-api]. Additional datasets used in this study, Camelyon16 [https://camelyon16.grand-challenge.org/], UBC-OCEAN [https://www.kaggle.com/competitions/UBC-OCEAN], and SICAPv2 [https://data.mendeley.com/datasets/9xxm58dvs3/1], were obtained from their respective public repositories.
Data analysis	All analyses were performed in a Python 3.10.4 environment created via venv, with core packages including PyTorch 1.12.1+cu113, TorchVision 0.13.1+cu113. Other dependencies were installed from a requirements.txt file using pip. The environment was set up on a system with GCCcore 11.3.0, CUDA 11.3.1, and cuDNN 8.2.1.32. OpenSlide 3.4.1 was used for handling whole-slide images. Using the custom code provided by us to do the data analysis. https://github.com/ZeyuGaoAi/SMMILe

For manuscripts utilizing custom algorithms or software that are central to the research but not yet described in published literature, software must be made available to editors and reviewers. We strongly encourage code deposition in a community repository (e.g. GitHub). See the Nature Portfolio guidelines for submitting code & software for further information.

Data

Policy information about availability of data

All manuscripts must include a data availability statement. This statement should provide the following information, where applicable:

- Accession codes, unique identifiers, or web links for publicly available datasets
- A description of any restrictions on data availability
- For clinical datasets or third party data, please ensure that the statement adheres to our policy

The TCGA datasets (TCGA-LU [including LUAD and LUSC], TCGA-RCC, and TCGA-STAD), including whole-slide images and diagnostic labels, are publicly available from the Genomic Data Commons portal (https://portal.gdc.cancer.gov). Public datasets, including whole-slide images and spatial annotations used in this study are available from their respective portals: Camelyon16 (https://camelyon16.grand-challenge.org/), UBC-OCEAN (https://www.kaggle.com/competitions/UBC-OCEAN), and SICAPv2 (https://data.mendeley.com/datasets/9xxm58dvs3/1). Processed spatial annotations, fine-grained subtype labels for TCGA-STAD, and annotations for TCGA-LU and TCGA-RCC are available at https://huggingface.co/datasets/zeyugao/SMMILe_SpatialAnnotation. Extracted patch embeddings, patch-level labels, and superpixel segmentation results for all six public datasets are deposited at https://huggingface.co/datasets/zeyugao/SMMILe_Datasets. Two in-house datasets (IH-RCC and IH-ESD) were collected with approval from the Ethics Committee of the First Affiliated Hospital of Xi'an Jiaotong University (KYLLSL2021-420 and KYLLSL2022-333). A waiver of informed consent was granted, and all samples were fully anonymized to remove identifiable information. The processed datasets (patch embeddings, labels, and superpixel segmentation results) are available at https://huggingface.co/datasets/zeyugao/SMMILe_PrivateDatasets under gated access. Access requests can be submitted via the repository page and will be reviewed and approved by Dr. Zeyu Gao (zg323@cam.ac.uk) within 4 weeks, subject to ethical compliance. The source data generated in this study are provided in the Source Data file.

Research involving human participants, their data, or biological material

Policy information about studies with human participants or human data. See also policy information about sex, gender (identity/presentation), and sexual orientation and race, ethnicity and racism.

Reporting on sex and gender	Our study focused exclusively on the analysis of whole-slide images (WSIs). While some TCGA cohorts include sex information that has been previously reported, we did not analyze it in this study because our research is centered on computational pathology and spatial quantification, which do not inherently involve sex- or gender-specific variables in imaging data. No clinical data were disclosed in the other public datasets. The in-house datasets that we collected also did not contain any clinical data beyond pathological diagnosis.
Reporting on race, ethnicity, or other socially relevant groupings	Our study focused solely on the analysis of whole slide images. Several TCGA cohorts contain race/ethnicity data that has been previously reported, but we did not analyse it. No clinical data were disclosed in the other public datasets. The in-house datasets that we collected also did not contain any clinical data beyond pathological diagnosis.
Population characteristics	Our study focused solely on the analysis of whole slide images. Several TCGA cohorts contain clinical data that has been previously reported, but we did not analyse it. No clinical data were disclosed in the other public datasets. The in-house datasets that we collected also did not contain any clinical data beyond pathological diagnosis.
Recruitment	For the IH-RCC dataset, our retrospective experiment included patients who had been diagnosed with primary renal cell carcinoma between 2012 and 2019. A maximum of 50 patients from each subtype were included, with multiple slides retained, such as the tumor center and junctions, all containing tissue from the primary tumor. We selected patients based on the timing of their diagnosis, from most recent to earliest, as in general, recent slides have better quality. For the IH-ESD dataset, our retrospective experiment involved a random selection of 44 patients (99 slides) who were clinically suspected of having early gastric cancer and underwent ESD surgical resection between 2016 and 2021. Each patient's complete set of tissue sections was retained for analysis. However, tissue sections from one patient were damaged and consequently, that patient was excluded from the study.
Ethics oversight	Two in-house datasets (IH-RCC and IH-ESD) were collected with IRB approval from the Ethics Committee of the First Affiliated Hospital of Xi'an Jiaotong University (KYLLSL2021-420, KYLLSL2022-333) for the current study, and there are no plans to make them publicly available.<br>The Ethics Committee of the First Affiliated Hospital of Xi'an Jiaotong University approved the waiver of informed consent. Patient data were anonymized to protect privacy, ensuring they contained no personal health information or identifiable markers.

Note that full information on the approval of the study protocol must also be provided in the manuscript.

Field-specific reporting

Please select the one below that is the best fit for your research. If you are not sure, read the appropriate sections before making your selection.

☒ Life sciences ☐ Behavioural & social sciences ☐ Ecological, evolutionary & environmental sciences

For a reference copy of the document with all sections, see nature.com/documents/nr-reporting-summary-flat.pdf

Life sciences study design

All studies must disclose on these points even when the disclosure is negative.

Sample size	The sample sizes for all public datasets used in this study (e.g., TCGA-LU, Camelyon16, UBC-OCEAN, SICAPv2) are fixed and widely adopted in prior research. These datasets have been extensively used and validated in computational pathology tasks, and their sample sizes have been shown to be sufficient for training and evaluating deep learning models. For the two in-house datasets (IH-RCC and IH-ESD), the number of whole slide images is of the same order of magnitude as the public datasets, with several hundred WSIs in each. Although no formal sample size calculation was performed, we consider the sample sizes adequate for the goals of this study, as they ensure statistical robustness and enable fair comparisons with prior benchmark studies.
Data exclusions	We excluded samples with damaged WSI original files (missing hierarchical information), poor WSI quality (non-standard staining, artificial contamination), and unclear diagnosis in the TCGA and In-house datasets.
Replication	Based on the code and datasets we provided and referenced, all the results are reproducible.
Randomization	For all experiments, we employed 5-fold cross-validation to estimate the predictive performance of each model. When patient identifiers were available, the splits were performed at the patient level to avoid data leakage. For datasets without patient-level metadata, the splits were conducted randomly at the WSI level. In each fold, four-fifths of the data were further split into training and validation sets (90%-10%), and the remaining fifth was used as the test set.
Blinding	In our study, which focuses on the analysis of pathological images, blinding to group allocation during data collection and/or analysis was not applicable. This is because our research methodology relies on automated computational models to analyze and interpret image data, independent of any subjective human assessment. Consequently, there is no requirement for blinding as the image processing and subsequent analysis are conducted algorithmically, based on predefined criteria and without investigator bias that would typically necessitate blinding. Thus, blinding was not relevant to the design and execution of our study.

Reporting for specific materials, systems and methods

We require information from authors about some types of materials, experimental systems and methods used in many studies. Here, indicate whether each material, system or method listed is relevant to your study. If you are not sure if a list item applies to your research, read the appropriate section before selecting a response.

Materials & experimental systems

n/a	Involved in the study
☒	☐ Antibodies
☒	☐ Eukaryotic cell lines
☒	☐ Palaeontology and archaeology
☒	☐ Animals and other organisms
☒	☐ Clinical data
☒	☐ Dual use research of concern
☒	☐ Plants

Methods

n/a	Involved in the study
☒	☐ ChIP-seq
☒	☐ Flow cytometry
☒	☐ MRI-based neuroimaging

Plants

Seed stocks	*Report on the source of all seed stocks or other plant material used. If applicable, state the seed stock centre and catalogue number. If plant specimens were collected from the field, describe the collection location, date and sampling procedures.*
Novel plant genotypes	*Describe the methods by which all novel plant genotypes were produced. This includes those generated by transgenic approaches, gene editing, chemical/radiation-based mutagenesis and hybridization. For transgenic lines, describe the transformation method, the number of independent lines analyzed and the generation upon which experiments were performed. For gene-edited lines, describe the editor used, the endogenous sequence targeted for editing, the targeting guide RNA sequence (if applicable) and how the editor was applied.*
Authentication	*Describe any authentication procedures for each seed stock used or novel genotype generated. Describe any experiments used to assess the effect of a mutation and, where applicable, how potential secondary effects (e.g. second site T-DNA insertions, mosiacism, off-target gene editing) were examined.*
